# Phylogenetic Accuracy Under Non-stationary and Non-homogeneous Conditions: A Simulation Study

**DOI:** 10.1093/sysbio/syag010

**Published:** 2026-02-05

**Authors:** Suha Naser-Khdour, Bui Quang Minh, Robert Lanfear

**Affiliations:** Department of Ecology and Evolution, Research School of Biology, 46 Sullivan's Creek Road, Australian National University, Canberra, Australian Capital Territory, ACT 2601, Australia; Department of Cell and Molecular Biology, Uppsala biomedicinska centrum (BMC), Husargatan 3, Uppsala University, Uppsala, Sweden; School of Computing, 108 North Road, Australian National University, Canberra, Acton ACT 2601, Australia; Department of Ecology and Evolution, Research School of Biology, 46 Sullivan's Creek Road, Australian National University, Canberra, Australian Capital Territory, ACT 2601, Australia

**Keywords:** Evolution under non-SRH conditions, model violations, phylogenetic inference, simulations, systematic bias

## Abstract

Phylogenetic inference typically assumes that the data have evolved under stationary, reversible, and homogeneous (SRH) conditions. Many empirical and simulation studies have shown that assuming SRH conditions can lead to significant errors in phylogenetic inference when the data violate these assumptions. Yet, many simulation studies focused on extreme non-SRH conditions that represent worst-case scenarios and not the average empirical data set. In this study, we simulate data sets under various degrees of non-SRH conditions using empirically derived parameters to mimic real data and examine the effects of incorrectly assuming SRH conditions on inferring phylogenies. Our results show that maximum likelihood inference is generally quite robust to a wide range of SRH model violations but is inaccurate under extreme convergent evolution.

Markov models are commonly used in model-based phylogenetic analyses such as maximum likelihood (ML) and Bayesian inference (Felsenstein 2004; Yang 2006). A Markov model is often represented by an instantaneous rate matrix Q of size 4-by-4 for DNA or 20-by-20 for protein sequences that describes the substitution rates between nucleotides or amino acids (henceforth denoted as states), respectively. The assumption of evolution under Markovian conditions is convenient because the probabilities of the next states only depend on the current states, independently of how the current states had evolved (Felsenstein 1981, 1983; Yang 1994; Swofford et al. 1996; Yang 2006). Such approaches can be made more mathematically and computationally tractable by assuming that the Markov model is stationary, reversible, and homogeneous (SRH), assumptions that are universal to many of the most commonly used models in phylogenetics such as those in the GTR (General Time Reversible) family (Kimura 1980; Felsenstein 1981; Hasegawa et al. 1985; Tavaré 1986; Tamura and Nei 1993; Yang 1994). Time homogeneity means that a single Q-matrix operates along all edges of the tree (i.e., all substitution rates stay constant through time). Stationarity means that the state frequencies also remain constant along all edges of the tree. Reversibility means that, at equilibrium, the rate of substitution from one state to another is equal to the rate of substitution in the reverse direction.

The assumptions of homogeneity, stationarity, and reversibility imply a potential departure from biological reality (Yang and Roberts 1995; Foster and Hickey 1999; Foster 2004; Ababneh et al. 2006). Various evolutionary processes, including non-stationary or non-reversible mutational processes, compositional heterogeneity driven by factors such as GC-biased gene conversion, and changes in selective pressures leading to amino acid compositional shifts, can impact phylogenetic inference when performed under the assumption of stationarity, reversibility, and homogeneity.

Hypermutations, such as the CpG islands in mammals (Bird 1980), is an example of a non-reversible model of evolution. Reversibility assumption implies that the likelihood of a tree will be the same regardless of the placement of the root (Felsenstein 1981). Moreover, a reversible substitution model has up to 8 free rate parameters for nucleotides and 208 for amino acids, whereas a non-reversible substitution model has up to 11 free rate parameters for nucleotides and 379 for amino acids, provided that the model is still stationary (Yang 1994). These degrees of freedom can increase dramatically if the model is non-stationary or/and non-homogeneous (Jayaswal et al. 2005, 2011a, 2011b, 2014) at the limit there can be an independent model of evolution on every branch of a tree, meaning that the total number of parameters is the product of the number of parameters in the substitution model and the number of branches in the tree.

Using stationary, reversible, and homogeneous substitution models to infer a phylogeny from data that has evolved under more complex conditions compromises the consistency of the ML estimation (Felsenstein 2004). Ideally, we would like to use data that comply with the assumptions of the models we apply, or alternatively, use models that are not violated by the data in hand. However, the use of non-SRH models is computationally demanding and is often not practical for large data sets. On the other hand, removing data that do not comply with the SRH assumption, as suggested by Jermiin et al. (2017), will come at the cost of losing phylogenetic information. Both studies of simulated data (Huelsenbeck and Hillis 1993; Hillis et al. 1994; Galtier and Gouy 1998; Conant and Lewis 2001; Ho and Jermiin 2004; Jermiin et al. 2004; Boussau and Gouy 2006) and empirical data (Galtier and Gouy 1995, 1998; Phillips et al. 2004; Collins et al. 2005; Jayaswal et al. 2005, 2007, 2011b; Phillips et al. 2010; Nguyen et al. 2012; Betancur-R et al. 2013; Naser-Khdour et al. 2019) have shown that applying SRH models to data that have evolved under more complex conditions may lead to significant errors in phylogenetic inference. However, most of these simulation studies have used parameters or parameter values that are not likely to reflect most empirical data sets, and sometimes represent extreme conditions such as the independent convergence of distantly related taxa to a GC content that differs substantially from the rest of the taxa in the tree (e.g., Hillis et al. 1994; Ho and Jermiin 2004; Jermiin et al. 2004). Although these simulations are based on biological observations such as the evolution of extreme GC content differences among closely related bacteria (Mooers and Holmes 2000), they do not represent the degree of violation of SRH conditions typical of most data sets. Indeed, apart from extreme cases (e.g., Jermiin et al. 2004) it remains relatively poorly understood to what extent different types and degrees of violations of the SRH conditions affect phylogenetic inference.

In this study, we examine the influence of violating the SRH assumptions on phylogenetic inference with SRH models using parameters and parameter values that are derived from thousands of empirical data sets. We simulate nucleotide alignments under various non-stationary (and thus non-reversible) or/and non-homogeneous conditions and examine the effects of incorrectly assuming SRH conditions on inferring phylogenies from these data. It is noteworthy that by non-homogeneous conditions we refer to heterogeneity across lineages and not heterogeneity across sites. In contrast to heterogeneity across lineages that are widely ignored in phylogenetic analyses, heterogeneity across sites is usually accounted for by using partitioning methods or a pre-defined distribution of rate variation among sites, such as Gamma distribution (Yang 1994). For the purpose of this study, we assume a constant rate of substitution among sites, and our simulations do not examine the effects of unmodeled site rate variation.

## Materials and Methods

### Code and Data Availability

All scripts and code used to perform the analyses in this study are available on GitHub at: https://github.com/suhanaser/empiricalGTRdist.

Simulated sequence alignments and associated metadata are deposited in the Dryad Digital Repository: https://doi.org/10.5061/dryad.k3j9kd582.

### Simulations

In order to investigate the ability of SRH models to correctly infer topologies and branch lengths from non-SRH data, we devised a new approach that allows us to simulate alignments gradually ranging from true SRH conditions (with identical base frequencies and identical reversible substitution processes on every branch of the topology) to the most extreme violation with completely unrelated base frequencies and non-reversible substitution processes on every branch of the topology. For an alignment of *m* taxa and *n* sites, we will denote the set of all branches in the rooted tree τ as ${\mathrm{\Phi }} = \{ {1,.\ .\ .,l} \}$.

We simulate data under two different simulation schemes as follows:

Scheme 1. An inheritance scheme (autocorrelation) designed to reflect the evolutionary process, in which each node in the tree inherits its substitution processes from its parent with a constant strength of inheritance modified by the branch length connecting the two nodes. The scheme reflects the continuity of evolutionary processes that are changing through time along a phylogenetic tree. The code used to perform these simulations is available in the inheritance_Simulations.py script of our GitHub repository.
Scheme 2. A two-matrix scheme designed to reflect previous approaches to simulating non-SRH evolution, where two independent subtrees (that are not sisters nor descendants of each other) have an identical substitution process that is distinct from the substitution process that operates on the rest of the tree. This scheme resembles convergent evolution. The code used to perform these simulations is available in the two_matrix_Simulations.py script of our GitHub repository.

Applying these two schemes allows us to ask how evolutionarily inspired non-SRH simulations are affected by SRH assumptions (scheme 1) and then to directly compare these with the more extreme forms of non-SRH evolution that are more often simulated (scheme 2). We will describe both simulation approaches in more detail below. But we start by describing how we choose model parameters for our simulations.

### Estimating Empirical Parameter Distributions and Tree Topologies for Simulations

Both of our simulation approaches require us to choose base frequency vectors and rate matrices with which to simulate alignments. Generating these at random could limit the applicability of our results because it is unlikely that randomly generated base frequency vectors or rate matrices would reflect reality. To address this, we instead estimated base frequency vectors and rate matrices from a large collection of empirical alignments and then used these parameters for our simulations.

In order to estimate the distributions of the empirical base frequencies (${\mathrm{\Pi}}$) and the substitution rates (*X*; often referred to as the rate matrix R, Yang 2006), we used 32,666 partitions from 49 nucleotide data sets (Supplementary Table A.1). These consist of different lengths (one site to over 23 Kb), different kingdoms (animals, plants, fungi, and archaea), different types of data (codon positions, rRNA, tRNA, introns, intergenic spacers, and UCEs), and different types of genomes (nuclear, mitochondria, virus, and plastid). Because different partitions of the genome evolve differently, for each partition, we ran IQ-TREE (Minh et al. 2020) with a GTR model and free rate heterogeneity across sites (Yang 1995) with four categories + invariant sites and extracted the parameters using the script extract_GTR_parameters_dist_from_empirical_data.py available on our GitHub repository. This gave us the distributions of 32,666 estimates of each parameter in the GTR matrix (A↔C, A↔G, A↔T, C↔G, C↔T, G↔T) and the distribution of each base frequency (${{\pi }_A}$, ${{\pi }_C}$, ${{\pi }_G}$, ${{\pi }_T}$).

We use a similar approach to estimate the distribution of branch lengths. Estimating branch lengths from each partition separately could be misleading because there tends to be a high stochastic error in branch lengths estimated from short single-partition alignments (Kumar et al. 2012). Therefore, in order to estimate the empirical distribution of the branch lengths, we instead estimated a separate phylogeny and corresponding branch lengths for each of our 49 (concatenated) nucleotide data sets and 18 amino acid data sets and extracted the empirical branch lengths from each phylogeny. For each data set, we ran IQ-TREE with the best-fit fully partitioned model (Chernomor et al. 2016), which allows each partition to have its own evolutionary model and edge-linked rates determined by ModelFinder (Kalyaanamoorthy et al. 2017). We then rooted the tree with the outgroup taxa (if provided) and extracted the empirical branch lengths of the ingroup (*T*) for each of the 33,178 partitions from 67 nucleotide and amino acid data sets.

Finally, for each parameter in *X* (5 parameters—G↔T equals to 1) and ${\mathrm{\Pi}}$ (4 parameters), and for each distribution in *T* (67 distributions—each data set is an independent distribution), we find the best-fit distribution from 36 common probability distributions using the Kolmogorov–Smirnov test using SciPy (Virtanen et al. 2020). Assuming that sampling among each distribution independently will approximate a sample from the joint distribution of parameters, we then sampled parameters for our simulations from these best-fit distributions. Because the parameters of *Π* are not independent, to sample a base-frequency vector we randomly sampled a parameter from each of the four base frequency’s best-fit distribution and then normalized these parameters to sum to 1.

The tree topology τ is derived from birth–death simulations with speciation rate λ, extinction rate μ, and the fraction of sampled taxa *f* using TreeSim package with a fixed number of extant species (Stadler 2011) using the run birth-death.R available on out GitHub repository. In principle, it is possible to estimate the speciation and extinction rates from empirical data (Nee et al. 1994; Rannala and Yang 1996; Magallon and Sanderson 2001). However, not knowing the fraction of sampled taxa a priori will tend to bias such estimates (Stadler 2013; Hua and Lanfear 2018). Because of the challenges of reliably estimating empirical speciation and extinction rates, we instead randomly sampled the speciation rate, the extinction rate, and the fraction of sampled taxa from uniform distributions, to attempt to cover all the realistic regions of the parameter space.


\begin{eqnarray*}
{\boldsymbol{\lambda }}\sim{\boldsymbol{U}}\left( {0,1} \right),{\mathrm{\ \ \ \ \ }}{\boldsymbol{\mu }}\sim{\boldsymbol{U}}\left( {0,{\boldsymbol{\lambda }}} \right),{\mathrm{\ \ \ \ \ }}{\boldsymbol{f}}\sim{\boldsymbol{U}}\left( {0,1} \right)
\end{eqnarray*}

Note that under these conditions λ is always greater than μ.

We simulated data sets with 20, 40, 60, 80, and 100 taxa. For each number of taxa (*m*), we simulated 3960 topologies. This number is derived from the total set of unique parameter combinations, which is explicitly detailed in the *Simulation Parameters* subsection below. For each of these simulations, the tree topology was generated using a birth–death process with random speciation rate (λ), random extinction rate (μ), and random fraction of sampled taxa (*f*). The branch lengths generated by this process were discarded and replaced with branch lengths randomly sampled from an arbitrary distribution from set *T* (2*m*–2 branch lengths in total).

Other Python libraries that we used for the simulations are NumPy (Walt et al. 2011), pandas (McKinney 2010), and ETE3 (Huerta-Cepas et al. 2016). The python scripts for all simulations can be found on GitHub (https://github.com/suhanaser/empiricalGTRdist).

### Inheritance Evolution: Inheritance Scheme Simulations

An evolutionary scenario would, ideally, have each lineage inheriting the parameters of its molecular evolutionary process from its parent lineage. At one extreme—where inheritance is perfect and the original evolutionary process is SRH—such a process would define a molecular evolutionary process that is SRH across the entire topology by simply defining a single SRH model at the root node. At the other extreme, where the association between parent and offspring lineages is no better than random and the original process is not SRH, there is no association between parent and offspring lineages and the process is maximally non-SRH. To mimic this situation, we designed a simulation approach that allows us to vary the homogeneity and stationarity assumptions both independently and together.

Our inheritance scheme allows us to vary the degree to which a single alignment has evolved under SRH conditions by simply adjusting the strength of inheritance of the substitution process and the base frequencies either jointly via a parameter we call *ρ *, or independently via parameters *ω * and *v*, respectively. When the inheritance parameters are set to 1 and the model at the root of the tree is reversible, the model will conform to SRH conditions. We can simulate increasing violation of SRH conditions simply by decreasing the inheritance parameters toward zero. When the relevant inheritance parameter is less than one, each branch inherits some proportion of its substitution model from the parent branch, whereas the remaining proportion of the model is selected at random from the empirical parameter distributions. In practice, the parameter in a descendant branch is calculated as the weighted sum of the parameter in the parent branch (where the weight is the inheritance parameter) and a randomly generated parameter from the appropriate empirical distribution (where the weight is one minus the inheritance parameter).

We simulated data under five different categories of conditions using this scheme, in order to examine independently and together the effects of relaxing the stationarity and homogeneity assumptions.

1. *SRH conditions (Fig. 1a)*.—In the simplest case for a model that conforms to the SRH assumptions, where model parameters are generated from the empirical distributions. This describes a model in which all branches inherit this reversible model from their parent branch without variation, such that all branches on the tree have the same reversible substitution model, conforming to the SRH assumptions.
2. *Relaxing the stationarity assumption (Fig. 1b)*.—In order to hold the homogeneity assumption but relax the stationarity assumption, we introduce a parameter called *ν * ($0 \le \nu \le 1$) that allows the state frequency to vary at the root while still keeping the same rate matrix for all branches of the tree. Mathematically, this can be described as
  \begin{eqnarray*}
{{Q}_i} = {{Q}_0} = {{\pi }_0}{{S}_0}\ \ \ \ \ \ \ \ \ \ \ \ \ \ \ \ \ \ \ \ \ \ \ i\in\left\{ {\mathrm{\Phi }} \right\}
\end{eqnarray*}
   
  \begin{eqnarray*}
\begin{array}{@{}*{1}{c}@{}} {\ \ \ \ \ \ \ \ \ \ \ \ \ \ \ \ \ \ \ }\\ {{{\pi }_{\mathit{root}}} = {{\nu }^{{{d}_{\mathit{root}}}}}{{\pi }_0} + \left( {1 - {{\nu }^{{{d}_{\mathit{root}}}}}} \right)\pi \ \ \ \ \ \{ \nu \in {\mathbb{R}}:0 \le \nu < 1\} \ } \end{array}
\end{eqnarray*}
  where ${{Q}_i}$ is the substitution rate matrix operating on branch *i*, ${{\pi }_0}$ is the stationary base frequencies, ${{\pi }_{\mathit{root}}}$ is the base frequencies at the root, and ${{d}_{\mathit{root}}}$ is the branch length of the root branch, which determines the probability of parameter inheritance. As ${{d}_{\mathit{root}}}$ increases, the exponential term ${{\nu }^{{{d}_{\mathit{root}}}}}$​ decreases, causing the base frequencies (${{\pi }_{\mathit{root}}}$) to decay exponentially toward the general stationary frequencies (π). When ${\mathrm{\nu }} = 1$, ${{\pi }_{\mathit{root}}}$ is equal to ${{\pi }_0}$ and this scheme boils down to the first SRH condition. When ${\mathrm{\nu }} = 0$, ${{\pi }_{\mathit{root}}}$ is equal to *π *, meaning that the root frequency is generated separately from ${{\pi }_0}$. ${{\pi }_{\mathit{root}}}$ will vary between these two extremes when ${\mathrm{\nu }}$ is between 0 and 1, with lower ${\mathrm{\nu }}$ reflecting a larger deviation from stationary conditions.
  Note that we denote by *S* the set of exchangeability parameters that define the substitution rate matrix *Q*, used in simulations and model fitting. In contrast, we use X to denote empirical substitution rates (e.g., A↔C, A↔G, A↔T, C↔G, C↔T, G↔T) that are directly estimated from observed alignments or derived from empirical substitution matrices.
3. *Relaxing the homogeneity assumption (Fig. 1c)*.—In order to hold the stationarity assumption but relax the homogeneity assumption we need to simulate data in which *ν * is set to 1 (such that all branches have the same base frequencies as the root node), but we introduce a parameter *ω * that varies between zero and one (such that the inheritance of the parameters of the *Q*-matrix ranges from completely random to near-perfect). We can describe this mathematically as follows:
  \begin{eqnarray*}
{{Q}_i} = \ {{\omega }^{{{d}_i}}}{{\pi }_0}{{S}_j} + \left( {1 - {{\omega }^{{{d}_i}}}} \right){{\pi }_0}S \\
i,j\in\left\{ {\mathrm{\Phi }} \right\},{\mathrm{\ \ \ }}\left\{ {\omega \in\mathbb{R}:0 \le \omega < 1} \right\}\
\end{eqnarray*}
   
  \begin{eqnarray*}
{{\pi }_{\mathit{root}}} = {{\pi }_{0\ }}
\end{eqnarray*}
  where ${{Q}_i}$ is the process operating on branch *i*, ${{S}_j}$ are the substitution rates on the parent branch of branch *i*, and ${{d}_i}$ is the branch length of the branch *i*.
4. *Relaxing the stationarity and homogeneity assumptions simultaneously but independently (Fig. 1d)*.—We can simulate non-stationary and non-homogeneous data by setting both *ν * and *ω * to values less than one. When we relax both assumptions, we will allow ${{Q}_i}$ and ${{\pi }_{\mathit{root}}}$ to vary simultaneously but independently:
  \begin{eqnarray*}
{{Q}_i} = \ {{\omega }^{{{d}_i}}}{{\pi }_0}{{S}_j} + \left( {1 - {{\omega }^{{{d}_i}}}} \right){{\pi }_0}S\ \ \ \ \ \ \ \ \ \ \ i\in\left\{ {\mathrm{\Phi }} \right\}, \\
{\mathrm{\ \ \ }}\left\{ {\omega \in\mathbb{R}:0 \le \omega < 1} \right\}\
\end{eqnarray*}
   
  \begin{eqnarray*}
{{\pi }_{\mathit{root}}} = {{\nu }^{{{d}_{\mathit{root}}}}}{{\pi }_0} + \left( {1 - {{\nu }^{{{d}_{\mathit{root}}}}}} \right)\pi \\
\left\{ {\nu \in\mathbb{R}:0 \le \nu < 1} \right\}
\end{eqnarray*}
5. *Relaxing the stationarity and homogeneity assumptions jointly (Fig. 1e)*.—Although the fourth set of simulation conditions, above, allows us to vary homogeneity and stationarity jointly but independently, it suffers from the limitation that we have a maximum of two base frequency vectors in the tree (${{\pi }_{\mathit{root}}}$ and ${{\pi }_0}).\ $ To relax this assumption further, we will allow ${{Q}_i}$ to vary while ${{\pi }_{\mathit{root}}}$ stays fixed. In those settings, both homogeneity and stationarity will increase with *ρ *.
  \begin{eqnarray*}
{{Q}_i} = \ \rho {{\pi }_{\mathrm{j}}}{{S}_{\mathrm{j}}} + \left( {1 - \rho } \right)\pi S{\mathrm{\ \ \ \ \ }}i\in\left\{ {\mathrm{\Phi }} \right\}, \\
{\mathrm{\ \ \ }}\left\{ {\rho \in\mathbb{R}:0 \le \rho \le 1} \right\}\ \
\end{eqnarray*}
   
  \begin{eqnarray*}
{{\pi }_{\mathit{root}}} = {{\pi }_{0\ }}
\end{eqnarray*}

**Figure 1.**
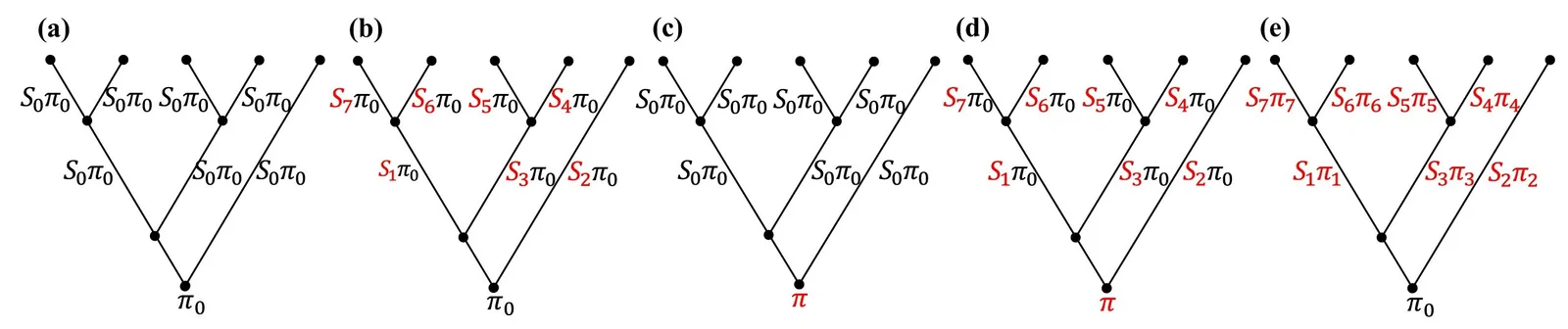
An example of five-taxon tree with different degrees of non-homogeneity and non-stationarity: (a) stationary and homogeneous, (b) stationary but not homogeneous, (c) non-stationary but homogeneous, (d) non-stationary and non-homogeneous, where the stationarity and homogeneity assumptions are relaxed simultaneously but independently, and (e) non-stationary and non-homogeneous, where the stationarity and homogeneity assumptions are relaxed jointly.

### Convergent Evolution: The Two-Matrix Scheme Simulations

Previous studies for simulating non-SRH evolution on phylogenies have used an approach in which two distantly related branches undergo severe but correlated changes in the molecular evolutionary process. To compare this approach with the more evolutionarily motivated approach described above, we randomly chose two nodes that are not sisters and not descendants of each other and assigned a different rate matrix (denoted by ${{S}_1}{{\pi }_1}$) from the rest of the tree to all their descendant branches (Fig. 2). The difference between the two matrices, Δ(Q), was measured using the Euclidean distance, which is calculated as the square root of the sum of the squared differences of the elements between the two Q-matrices. Specifically, we treated each Q-matrix as a vector of nine independent rate parameters (six substitution rates and three independent base frequencies) and calculated the distance between these nine-dimensional vectors.

**Figure 2.**
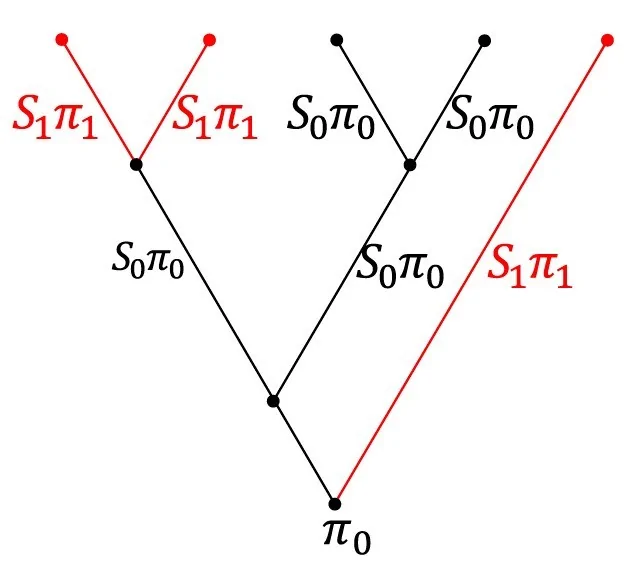
Non-stationary and non-homogeneous process with two different rate matrices ${{{\mathrm{Q}}}_0}$ and ${{{\mathrm{Q}}}_1}$.

### Simulation Parameters

The simulation parameters that we use in this study are the strength of inheritance of the substitution process (*ω *), strength of inheritance of the base frequencies (*ν *), strength of inheritance of the substitution process and base frequencies (*ρ *), number of sites (*n*), and number of taxa (*m*), where the parameter space is


\begin{eqnarray*}
\omega ,\nu ,\ \rho \in \left\{ 0,{{{10}}^{ - 25}},{{{10}}^{ - 20}},{{{10}}^{ - 15}},{{{10}}^{ - 10}},{{{10}}^{ - 5}}, \right. \\
\left. {{{10}}^{ - 4}},{{{10}}^{ - 3}},{{{10}}^{ - 2}},0.1,1.0 \right\}
\end{eqnarray*}

\begin{eqnarray*}
n\in\left\{ {100,1000,\ 10000} \right\}
\end{eqnarray*}

\begin{eqnarray*}
m\in\left\{ {20,\ 40,\ 60,\ 80,100} \right\}
\end{eqnarray*}

The inheritance weight parameters (ω$,\nu ,\rho $) were chosen to represent an even spread of the probability that a lineage retains its ancestral substitution process. This probability is calculated by raising the inheritance weight parameter to the power of the branch length (*d*). This models an exponential decay in the probability of inheritance as a function of the evolutionary distance. The number of taxa and number of sites are chosen to reflect typical sizes of empirical data sets. For simulation under the inheritance scheme, we simulated 10 alignments of each combination of *n, m*, $\ \nu $, and *ω * (1815 combinations) or *n, m*, and *ρ * (165 combinations) for a total of 19,800 simulations, or 3960 simulations for each of the five numbers of taxa in *m*.


\begin{eqnarray*}
&& \left( {11\ \left( {\omega ) \times 11\ (\nu } \right) + 11\ \left( \rho \right)} \right){\mathrm{\ values}} \times 3\ {\mathrm{No}}.{\mathrm{\ sites}}\ \left( n \right) \\
&& \quad \times \,\, 5\ {\mathrm{No}}.{\mathrm{\ taxa}}(m) \times 10\ {\mathrm{replicates}}
\end{eqnarray*}

For simulation under the two-matrix scheme, we simulated 1000 alignments of each combination of *n* and *m* for a total of 15,000 simulations.


\begin{eqnarray*}
3\ {\mathrm{No}}.{\mathrm{\ sites}}\ \left( n \right) \times 5\ {\mathrm{No}}.{\mathrm{\ taxa}}\left( m \right) \times 1000\ {\mathrm{replicates}}
\end{eqnarray*}

### Tree Inference

Our first goal is to understand how the incorrect use of SRH models on data that have evolved under non-SRH processes can affect phylogenetic inference. To do this, we compare the tree topologies and branch lengths estimated with SRH models in IQ-TREE to the topologies and branch lengths used to simulate each data set. For each simulated alignment, we ran IQ-TREE with ModelFinder (Kalyaanamoorthy et al. 2017) and 1000 ultrafast bootstrap replicates (Hoang et al. 2018). In order to assess the ability of SRH models to infer the correct tree topology, we then compared the simulated tree topology to the estimated tree topology using three different metrics—Normalized Robinson–Foulds (NRF) distance, which measures the number of splits that are present in one tree but not the other normalized to vary between 0 and 1 (Robinson and Foulds 1981), quartet distance (QD), which measures the number of four-taxon subtrees (quartets) that differ between the two trees (Estabrook et al. 1985), and the Normalized Path Difference (NPD) distance, which measures the difference in the number of edges along the paths between all pairs of tips on the two trees (Steel and Penny 1993). In order to assess the accuracy of branch length estimates, we ran IQ-TREE with the fixed true topology and then calculated the coefficient of determination *R*^2^ between the true and estimated branch lengths.

## Results

### Empirical Distributions

We derived the empirical distributions of the substitution model parameters, the nucleotide frequencies, and the proportion of invariant sites from 32,666 nucleotide alignments (Supplementary Table A.2). The empirical distribution of branch lengths we derived from 67 nucleotide and amino acid alignments consisting of 33,178 partitions (Supplementary Table A.1).

Using the Kolmogorov–Smirnov test, we found the best-fit probability distribution for each one of these empirical distributions (Table 1; Supplementary Table A.2 and Supplementary Figs. A.1–A.3).

**Table 1.** The best-fit probability distribution by the Kolmogorov–Smirnov test

Parameter	Best-fit distribution	Shape (α)	Scale (β)	Location (${{{\boldsymbol{x}}}_0}$)
A↔C	Log-Laplace	1.695	1.636	−0.152
A↔G	Log-Laplace	1.465	4.930	−0.140
A↔T	Inverse-Weibull	3.015	1.841	−1.154
C↔G	Inverse-Weibull	1.793	1.651	−0.741
C↔T	Log-Laplace	1.551	5.182	−0.175
${{{\boldsymbol{\pi }}}_{\boldsymbol{A}}}$	Generalized-logistic	0.557	0.026	0.313
${{{\boldsymbol{\pi }}}_{\boldsymbol{T}}}$	Exponential-Weibull	0.843, 4.872	0.294	−0.001
${{{\boldsymbol{\pi }}}_{\boldsymbol{C}}}$	Exponential-normal	1.769	0.027	0.173
${{{\boldsymbol{\pi }}}_{\boldsymbol{G}}}$	Power-log-normal	0.090, 0.039	0.614	−0.471
%I	Beta	0.577, 4.707	2.162	−5.4e-31
Branch length	Power-log-normal	1.208, 1.443	0.017	−7.1 e-05
Internal	Log-normal	1.272	0.010	−1.5 e-04
Leaves	Power-log-normal	2.805, 1.838	0.076	3.1 e-05

Furthermore, we checked if the distributions of the internal branch lengths and the leaf branch lengths were different, as is frequently observed in empirical data sets. Our results show that the distribution of the leaf branch lengths is similar to the general distribution of all branches, but with different parameters. On the other hand, the distribution of the internal branch lengths is different (Supplementary Fig. A.3). Because these differences were minor and our focus is on the effects of model violations rather than tree shape itself, we will use the general branch length distribution for all branches.

### Both Simulation Schemes Capture the True Extent of Compositional Heterogeneity

In order to confirm that our simulation settings are producing compositional variance patterns that are of the same order of magnitude as what we observed in the empirical data, we compared the variance in GC content between the simulated and empirical data sets.

Our results confirm that the compositional variance in both simulation schemes is indeed similar to the empirical data (Fig. 3). Because ω and ν exhibit identical patterns, we only included ν in the figure.

**Figure 3.**
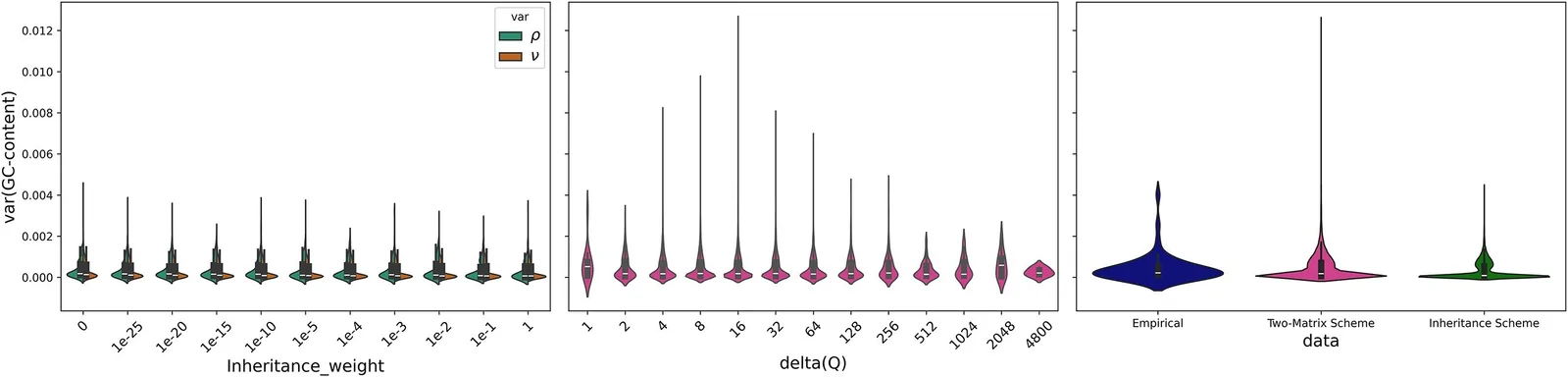
Variance in GC content in simulated and empirical data sets. The variance in GC content was calculated between sequences within each data set.

### Phylogenetic Inference Is Unaffected by Violation of SRH Conditions in an Inheritance Framework

Surprisingly, our results for the inheritance simulation scheme show that there is no detectable relationship between the severity with which SRH conditions were violated during the simulations and the accuracy of the tree topology or the tree length inferred from the simulated data. Specifically, we saw no relationship between the inheritance weight and the NRF (Normalized Robinson–Foulds), QD (quartet distance), or NPD (Normalized Path Difference) metrics in any of our inheritance simulations regardless of the tree length (Fig. 4; Supplementary Figs. A.4–A.7 and A.13). These metrics measure the difference between the inferred tree and the tree from which the alignment was simulated. If a stronger violation of the SRH conditions affects phylogenetic inference, we should expect to see that the distances are higher when the inheritance weight is lower, because a lower inheritance weight implies a stronger model violation through less homogeneity (for the rate matrix) and less stationarity (for the base frequencies). It is important to note that the results presented in Figure 4 represent averages across data sets with varying numbers of taxa and sites. Although Supplementary Figure A.5 represents the non-normalized RF metric, because the subfigures all have the same number of taxa, NRF and RF are essentially the same.

**Figure 4.**
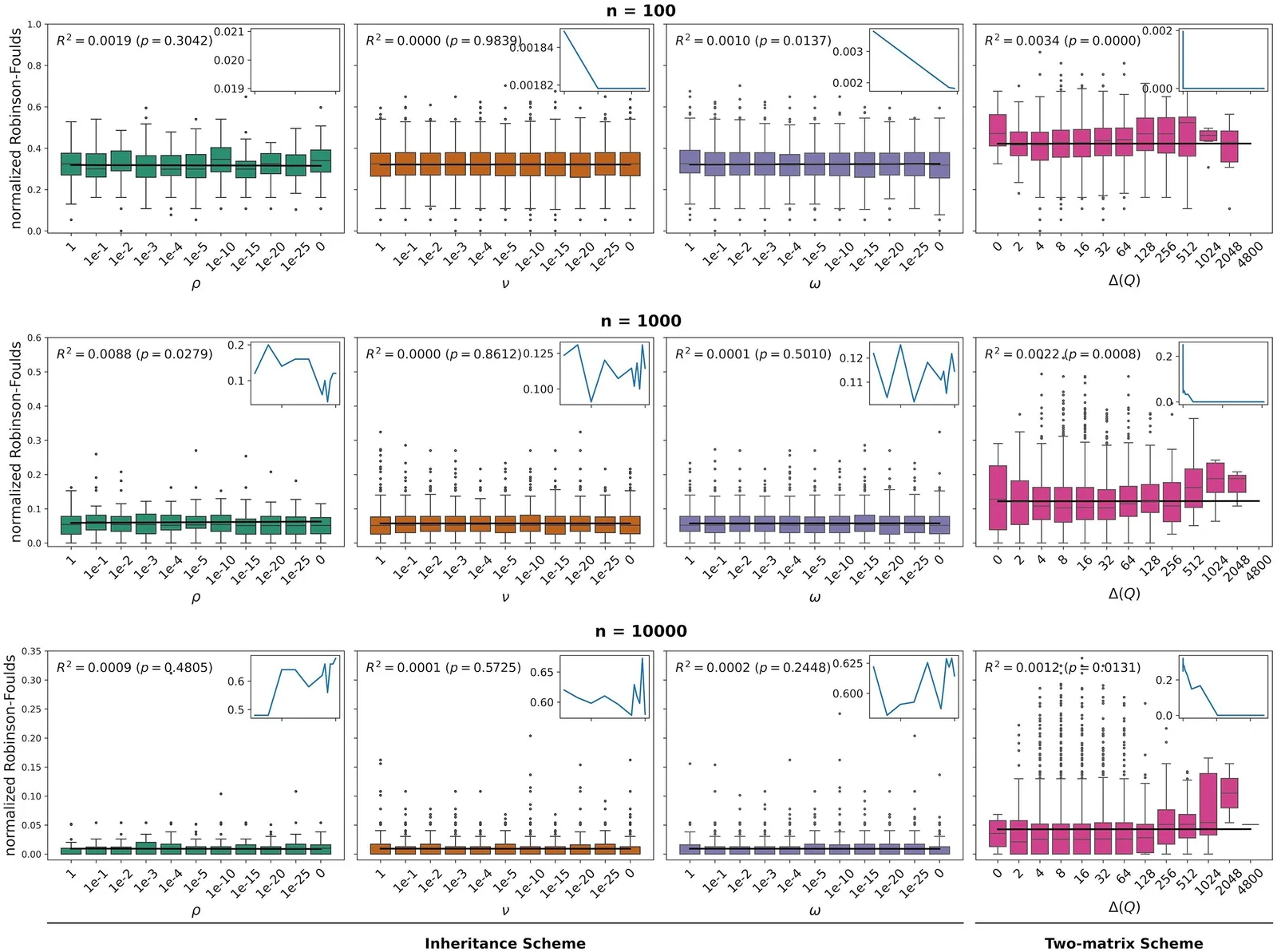
Normalized Robinson–Foulds distance between the estimated tree topology and the original tree topology as a function of the inheritance weight (ν, ω, ρ) in the first simulation scheme, and the distance between the two matrices (Δ(Q)) in the second simulation scheme, for different number of sites. The horizontal line on top of the boxplots represents the linear trend (regression) fitted to the data, which quantifies the strength of the monotonic relationship between the discrete ordered parameter values and the Normalized Robinson–Foulds (NRF) distance. The small plots show the proportion of data sets in which the distance between the estimated topology and the original topology equals zero as a function of the inheritance weight and the distance between the two matrices. The *x*-axis of each inset plot matches that of the bigger plot, labels have been excluded for clarity. If violation of SRH model assumptions increases topological error, we expect the NRF distance to increase toward the right of each plot. The figure shows that for the first simulation scheme, which mimics an inheritance evolutionary process, there is no detectable association between violation of SRH conditions and topological error. For the second simulation scheme, which mimics an extreme convergent situation, topological error increases with increasing violation of SRH conditions.

In addition, our results show that the proportion of simulated data sets for which the simulated tree is recovered from the simulated alignment is constant at around 0.25 in the inheritance scheme simulations regardless of the inheritance weight (Fig. 4; Supplementary Fig. A.4). Finally, we see that the branch length estimation, quantified by the coefficient of determination (*R*^2^) between the true and estimated branch lengths, demonstrate remarkable robustness across all parameters and violation levels, maintaining a mean *R*^2^ of 0.8 (median 0.9), suggesting that violation of SRH assumptions in our evolutionary framework has no detectable effect on the estimation of branch lengths (Fig. 5; Supplementary Fig. A.11).

**Figure 5.**
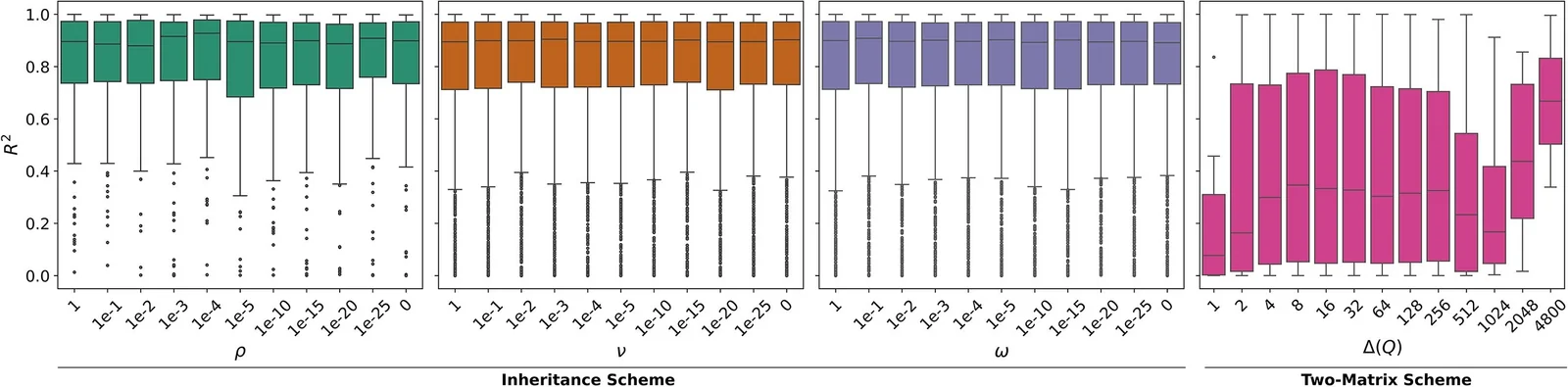
The accuracy of branch length estimation (*R*^2^) as a function of the inheritance weights (ν, ρ, ω) in the first simulation scheme, and the distance between the two matrices (Δ(Q)) in the second simulation scheme (right-hand panel).

### Tree Topologies and Branch Lengths Are Affected by Severe and Convergent Violation of SRH Conditions

Our results show that convergent violation of SRH assumptions by allowing two distantly related branches to have identical substitution models has increasingly severe effects on phylogenetic inference as the severity of the changes in the substitution models increases. Under the two-matrix scheme, we expect to see higher distances between the true tree and the estimated tree when there are larger Euclidean distances between the original matrix and the matrix under which the divergent clades evolve. In two out of the three metrics (Robison–Foulds and Path Difference), we found a weak but significant correlation between the distance between the matrices and the distance between the topologies (Fig. 4; Supplementary Figs. A.4 and A.8–A.10). However, in the third metric (QD) we found no correlation. Notably, the distance between the true tree and the estimated tree increases only when the Euclidean distance between the two matrices is very high. Nevertheless, the proportion of simulated data sets for which the simulated tree is recovered from the simulated alignment declines exponentially as the difference between the matrices in the two-matrix scheme increases (Fig. 4). This consistent lack of accuracy is observed regardless of the tree length (Supplementary  Fig. A.14).

The proportion of analyses in which the simulated tree is recovered declines from around 0.20 when there is no model violation to zero when the Euclidean distance between the matrices is around 2000, confirming that even the lowest levels of SRH violation have detectable negative effects on phylogenetic inference under the two-matrix scheme.

Finally, we see that the accuracy of branch length estimation is also severely impaired, with a mean *R*^2^ of 0.4 (median 0.3), suggesting that the structural error introduced by the convergent framework fundamentally degrades the recovery of true branch lengths (Fig. 5; Supplementary Fig. A.12).

## Discussion

Using two different simulation schemes, we explored the impact of violating the assumption of evolution under stationary, reversible, and homogeneous (SRH) conditions on ML phylogenetic tree inference. Our study extends the simulations in many previous studies by simulating data under an evolutionary scenario in which molecular evolutionary models evolve along a phylogeny. An interesting avenue for future research would be to separate overall tree length from the distribution of branch length proportions, which would allow for a more nuanced investigation of these parameters. Our results show that the inference of phylogenetic tree topologies and branch lengths are surprisingly robust to violations of SRH assumptions under an evolutionary scheme. But similarly to previous studies, we show that in extreme cases of convergent molecular evolution, the incorrect assumption of SRH conditions can severely mislead phylogenetic inference. In this study, we implemented two simulation schemes: one in which substitution processes are inherited and evolve along the tree, and a second in which two monophyletic clades evolve under an alternative process.

The first simulation scheme we introduced in this paper, which we called *the inheritance scheme*, allows tree branches to inherit their substitution process from their ancestor. The second simulation scheme, which we called *the two-matrix scheme*, is similar to previous studies and allows two distantly related monophyletic subtrees to evolve with a different evolutionary process from the rest of the tree (Galtier and Gouy 1995; Jermiin et al. 2004; Jayaswal et al. 2011a; Duchene et al. 2017).

Surprisingly, our results show no correlation between errors in the topology or branch length distribution inference and any of the inheritance scheme parameters, even in extreme cases where the evolutionary process is completely heterogeneous and non-stationary. These results indicate that ML tree inference with SRH models is surprisingly robust to even quite extreme violations of the SRH conditions.

It is important to note that, by holding the true tree topology fixed, we did not investigate how topological discordance—whether arising from estimation error or from discordance-inducing biological processes—interacts with branch length estimation biases (Mendes and Hahn 2016). In practice, the subset of branches whose inferred lengths are biased will depend on the nature and magnitude of topological error, which itself can be generated by SRH violations (e.g., like those examined here). A full assessment that varies topology and branch lengths jointly, that is, exploring both the discrete and continuous dimensions of phylogenetic space, remains an important avenue for future work on the consequences of SRH assumption violations.

Under the two-matrix simulation scheme, we found a small but significant increase in topological inference error and the extent of the violation of the SRH assumptions. Specifically, the more extreme the evolutionary convergence, the larger the errors in topological inference that assume SRH conditions. Moreover, we found that the accuracy of branch length estimation was severely degraded. These results emphasize the limitations of ML inference to operate under certain model violations, especially when these violations are highly imbalanced along the tree, as in the case of the two-matrix scheme simulations. These results indicate that the inference of the substitution model is more influenced by the imbalance of the model violation distribution along the tree than by the model violation itself. This conclusion agrees well with all previous simulation studies of similar simulation conditions (e.g., Conant and Lewis 2001; Jermiin et al. 2004; Duchene et al. 2017).

It is noteworthy that our results from the different simulation schemes agree with the results from empirical data (Naser-Khdour et al. 2019). When the model violations were more severe, like in the two-matrix scheme, we obtained a significant correlation between tree inference error and the extent of the violation of the SRH assumptions. In the inheritance scheme simulations, on the other hand, the violation of the SRH assumptions was milder, and therefore the phylogenetic inference was more robust. These results indicate that the extent of the violation of the SRH assumptions in the empirical data sets was higher than in our simulations under the inheritance scheme and it was probably closer to the extent of violation in the two-matrix scheme simulations.

Our findings suggest that although severe non-SRH evolution can be problematic, phylogenetic inference using standard ML is quite robust to the more realistic, minor violations present in many empirical data sets. However, we suggest reducing model violation by using more complex substitution models (e.g., Galtier and Gouy 1998; Tamura and Kumar 2002; Blanquart and Lartillot 2008; Dutheil et al. 2012; Zou et al. 2012; Groussin et al. 2013; Jayaswal et al. 2014) and using the new protocol of phylogenetic inference (Jermiin et al. 2020). This protocol emphasizes the importance of explicitly testing the assumptions of the evolutionary model before inference and using goodness-of-fit tests after inference to evaluate how well the model explains the data. By following this protocol, researchers can make more informed decisions about model choice and increase the accuracy of their final phylogenetic tree.

For the purpose of this study, in order to simulate data that mimic as closely as possible empirical alignments, we extracted the empirical distributions of base frequencies, substitution rates, the proportion of invariable sites, and branch lengths from tens of thousands of data subsets derived from 49 empirical nucleotide data sets and 18 empirical amino acid data sets. In addition to their use in this paper, these empirical distributions, along with their best-fit distributions, may be useful for a wide variety of simulation studies, or for specifying prior distributions for Bayesian phylogenetic methods.

We acknowledge that the empirically derived parameters we used as a basis for our simulations are not free of the stochastic and systematic errors inherent to phylogenetic inference on real data. For example, our branch length distributions may be affected by the hemiplasy that can occur when concatenating partitions with different underlying topologies (Mendes and Hahn 2016; Ogilvie et al. 2022). Moreover, in empirical data, many phylogenetic parameters are correlated, and our independent sampling approach does not fully capture this complexity. We believe those limitations of our study are an important area for future research. Yet, we also believe that our approach still represents a significant improvement over the use of arbitrary, theoretical parameter values, as it at least captures the broad range of empirical parameter distributions.

## Supplementary Material

syag010_Supplemental_File

## Data Availability

The Appendix, which contains all supplementary figures and tables, is available at Dryad Data Repository: https://doi.org/10.5061/dryad.k3j9kd582
