# Supplementary material for: Phylogenetic Accuracy Under Non-stationary and Non-homogeneous Conditions: A Simulation Study
Source: Syst Biol. 2026 Feb 5;75(6):1196–206. doi: 10.1093/sysbio/syag010 (PMC13637618; doi:10.1093/sysbio/syag010)
Supplement: Supplementary file 1 [file syag010_supplemental_file.pdf]

Appendix

TABLES

TABLE A.1. Number of Taxa, Number of Sites, Type, Clade, and Study Reference for Each Data Set That Have Been Used in This Study

	Dataset	Study Reference	Dataset Reference	Type	Clade	Taxa	Sites	Partitions	Tot. Tree Length	Avg. Branch Length
1	Anderson_2013	(Anderson, et al. 2014)	(Anderson, et al. 2013)	DNA	Loliginidae	145	3037	4	3.890	0.027
2	Ballesteros_2019	(Ballesteros and Sharma 2019a)	(Ballesteros and Sharma 2019b)	AA	Chelicerata	53	1484206	3534	11.966	0.226
3	Becker_2016	(Becker, et al. 2016)	(Becker, et al. 2017)	AA	Halobacteriacea	170	217	1	16.758	0.107
4	Bergsten_2013	(Bergsten, et al. 2013a)	(Bergsten, et al. 2013b)	DNA	Dytiscidae	38	2111	8	3.651	0.096
5	Borowiec_2015	(Borowiec, et al. 2015)	(Borowiec, et al. 2016)	AA	Metazoa	36	384981	1080	18.089	0.502
6	Branstetter_2017	(Branstetter, et al. 2017b)	(Branstetter, et al. 2017a)	DNA	Aculeata	187	183747	807	17.888	0.096
7	Broughton_2013	(Broughton, et al. 2013b)	(Broughton, et al. 2013a)	DNA	Osteichthyes	61	19997	61	5.870	0.096
8	Brown_2012	(Brown, et al. 2012b)	(Brown, et al. 2012a)	DNA	Ptychozoon	41	1665	7	14.416	0.352
9	Cannon_2016a	(Cannon, et al. 2016a)	(Cannon, et al. 2016b)	AA	Metazoa	78	44896	212	20.669	0.265
10	Cannon_2016b	(Cannon, et al. 2016a)	(Cannon, et al. 2016b)	DNA	Metazoa	78	89792	424	11.003	0.141
11	Chen_2015	(Chen, et al. 2015b)	(Chen, et al. 2015a)	AA	Gnathostomata	58	1806035	4682	3.482	0.060
12	Cognato_2001	(Cognato and Vogler 2001b)	(Cognato and Vogler 2001a)	DNA	Coleoptera: Scolytinae	44	1897	7	4.985	0.113
13	Crawford_2012	(Crawford, et al. 2012b)	(Crawford, et al. 2012a)	DNA	Sauria	10	465241	1145	0.242	0.024
14	Day_2013	Day, et al. (2013a)	(Day, et al. 2013b)	DNA	Synodontis	152	3586	11	1.424	0.009
15	Devitt_2013	(Devitt, Devitt, et al. 2013)	(Devitt, Cameron Devitt, et al. 2013)	DNA	Ensatina eschscholtzii klauberi	69	823	4	0.099	0.003
16	Dornburg_2012	(Dornburg, et al. 2012b)	(Dornburg, et al. 2012a)	DNA	Teleostei: Beryciformes: Holocentridae	44	5919	21	0.600	0.014
17	Faircloth_2013	(Faircloth, et al. 2013b)	(Faircloth, et al. 2013a)	DNA	Actinopterygii	27	149366	491	1.622	0.060
18	Fong_2012	(Fong, et al. 2012b)	(Fong, et al. 2012a)	DNA	Vertebrata	110	25919	168	3.130	0.028
19	Horn_2014	(Horn, et al. 2014b)	(Horn, et al. 2014a)	DNA	Euphorbia	197	11587	28	1.361	0.007
20	Irisarri_2017	(Irisarri, et al. 2017b)	(Irisarri, et al. 2017a)	AA	Gnathostomata	100	1964439	4593	3.795	0.038
21	Jarvis_2015	(Jarvis, et al. 2015)	(Jarvis, et al. 2014)	AA	Aves	52	4519041	8295	2.577	0.050
22	Kawahara_2013	(Kawahara and Rubinoff 2013a)	(Kawahara and Rubinoff 2013b)	DNA	Hyposmocoma	70	2238	9	1.745	0.025
23	Lartillot_2012	(Lartillot and Delsuc 2012b)	(Lartillot and Delsuc 2012a)	DNA	Eutheria	78	15117	51	3.817	0.049
24	Leache_2015	(Leache, et al. 2015)	(Leaché, et al. 2015)	DNA	Phrynosomatinae	11	358363	583	0.140	0.013

25	Looney_2016	(Looney, et al. 2016)	(Looney, et al. 2015)	DNA	Russula	1171	3927	4	16.156	0.014
26	McCormack_2013	(McCormack, et al. 2013b)	(McCormack, et al. 2013a)	DNA	Neoaves	33	539526	1541	0.356	0.011
27	Meiklejohn_2016	(Meiklejohn, et al. 2016a)	(Meiklejohn, et al. 2016b)	DNA	Phasianidae	18	614159	1501	0.113	0.006
28	Misof_2014	(Misof, et al. 2014b)	(Misof, et al. 2014a)	AA	Insecta	144	595033	2868	24.072	0.065
29	Moyle_2016	(Moyle, et al. 2016b)	(Moyle, et al. 2016a)	DNA	Oscines	106	375172	515	1.121	0.011
30	Murray_2013	(Murray, et al. 2013a)	(Murray, et al. 2013b)	DNA	Eucharitidae	237	3111	9	7.791	0.033
31	Near_2013	(Near, et al. 2013b)	(Near, et al. 2013a)	DNA	Acanthomorpha	608	8577	30	14.482	0.024
32	Nguyen_2016a	(Nguyen, et al. 2016b)	(Nguyen, et al. 2016a)	AA	Hymenoptera	17	688	1	0.819	0.048
33	Nguyen_2016b	(Nguyen, et al. 2016b)	(Nguyen, et al. 2016a)	AA	Hymenoptera	31	680	1	0.737	0.024
34	Nguyen_2016c	(Nguyen, et al. 2016b)	(Nguyen, et al. 2016a)	AA	Hymenoptera	25	811	1	0.529	0.021
35	Nguyen_2016d	(Nguyen, et al. 2016b)	(Nguyen, et al. 2016a)	AA	Hymenoptera	17	704	1	1.503	0.088
36	Nguyen_2016e	(Nguyen, et al. 2016b)	(Nguyen, et al. 2016a)	AA	Hymenoptera	17	385	1	1.246	0.073
37	Nguyen_2016f	(Nguyen, et al. 2016b)	(Nguyen, et al. 2016a)	AA	Hymenoptera	17	583	1	1.163	0.068
38	Oaks_2011	(Oaks 2011b)	(Oaks 2011a)	DNA	Crocodylia	79	7282	50	0.248	0.003
39	Prebus_2017	(Prebus 2017b)	(Prebus 2017a)	DNA	Temnothorax	50	1561581	2098	1.073	0.021
40	Pyron_2011	(Pyron and Wiens 2011)	(Pyron, et al. 2011)	DNA	Amphibia	2872	12712	34	106.062	0.037
41	Ran_2018a	(Ran, et al. 2018b)	(Ran, et al. 2018a)	AA	Spermatophyta	38	432014	1308	2.901	0.076
42	Ran_2018b	(Ran, et al. 2018b)	(Ran, et al. 2018a)	DNA	Spermatophyta	38	1296042	3924	4.091	0.108
43	Reddy_2017	(Reddy, et al. 2017b)	(Reddy, et al. 2017a)	DNA	Aves	235	137324	88	14.951	0.064
44	Richart_2015	(Richart, et al. 2016b)	(Richart, et al. 2016a)	DNA	Ischyropsalidoidea	6	536124	2016	7.249	1.208
45	Rightmyer_2013	(Rightmyer, et al. 2013b)	(Rightmyer, et al. 2013a)	DNA	Hymenoptera: Megachilidae	94	3692	25	4.599	0.049
46	Sauquet_2011	(Sauquet, et al. 2012)	(Sauquet, et al. 2011)	DNA	Nothofagus	51	5444	10	0.703	0.014
47	Seago_2011	(Seago, et al. 2011b)	(Seago, et al. 2011a)	DNA	Coccinellidae	97	2253	7	58.142	0.599
48	Sharanowski_2011	(Sharanowski, et al. 2011b)	(Sharanowski, et al. 2011a)	DNA	Braconidae	139	3982	11	8.595	0.062
49	Shen_2018	(Shen, et al. 2018)	(Shen 2018)	AA	Saccharomycotina	343	1162805	2407	68.137	0.100
50	Siler_2013	(Siler, Oliveros, et al. 2013)	(Siler, Brown, et al. 2013)	DNA	Lycodon	61	2697	7	1.757	0.029
51	Smith_2014	(Smith, et al. 2014b)	(Smith, et al. 2014a)	DNA	Xenops minutus	8	825804	1366	0.012	0.001
52	Tolley_2013	(Tolley, et al. 2013b)	(Tolley, et al. 2013a)	DNA	Chamaeleonidae	203	5054	16	17.324	0.085
53	Unmack_2013	(Unmack, et al. 2013b)	(Unmack, et al. 2013a)	DNA	Melanotaeniidae	139	6827	25	3.391	0.024
54	Varga_2019	(Varga, et al. 2019b)	(Varga, et al. 2019a)	DNA	Basidiomycota	5285	5737	3	153.916	0.029
55	Wainwright_2012	(Wainwright, et al. 2012a)	(Wainwright, et al. 2012b)	DNA	Acanthomorpha	188	8439	30	5.316	0.028
56	Whelan_2017	(Whelan, et al. 2017a)	(Whelan, et al. 2017b)	AA	Metazoa	76	49388	127	12.149	0.160
57	Wood_2012	(Wood, et al. 2013)	(Wood, et al. 2012)	DNA	Archaeidae	37	5185	8	10.735	0.335
58	Worobey_2014a	(Worobey, et al. 2014b)	(Worobey, et al. 2014a)	DNA	Influenzavirus A	146	1716	3	1.389	0.010

59	Worobey_ 2014b	(Worobey, et al. 2014b)	(Worobey, et al. 2014a)	DNA	Influenzavirus A	327	759	3	2.510	0.008
60	Worobey_ 2014c	(Worobey, et al. 2014b)	(Worobey, et al. 2014a)	DNA	Influenzavirus A	92	1416	3	0.805	0.009
61	Worobey_ 2014d	(Worobey, et al. 2014b)	(Worobey, et al. 2014a)	DNA	Influenzavirus A	355	1497	3	3.107	0.009
62	Worobey_ 2014e	(Worobey, et al. 2014b)	(Worobey, et al. 2014a)	DNA	Influenzavirus A	340	699	3	2.250	0.007
63	Worobey_ 2014f	(Worobey, et al. 2014b)	(Worobey, et al. 2014a)	DNA	Influenzavirus A	332	2151	3	2.885	0.009
64	Worobey_ 2014g	(Worobey, et al. 2014b)	(Worobey, et al. 2014a)	DNA	Influenzavirus A	326	2274	3	1.954	0.006
65	Worobey_ 2014h	(Worobey, et al. 2014b)	(Worobey, et al. 2014a)	DNA	Influenzavirus A	351	2280	3	3.279	0.009
66	Wu_2018a	(Wu, et al. 2018)	(Wu, et al. 2019)	AA	mammalia	90	3050199	5162	4.388	0.049
67	Wu_2018b	(Wu, et al. 2018)	(Wu, et al. 2019)	DNA	mammalia	90	9150597	15486	5.346	0.059

TABLE A.2. The probability distributions and their probability density function that we used for the Kolmogorov-Smirnov test.

distribution	PDF	notes
Alpha	$\frac{1}{x^2\Phi(\alpha)\sqrt{2\pi}} e^{-\frac{1}{2}(\alpha-\frac{1}{x})^2}$	Φ is the normal CDF<br>α is the shape parameter
Beta	$\frac{\Gamma(\alpha+\beta)x^{\alpha-1}(1-x)^{\beta-1}}{\Gamma(\alpha)\Gamma(\beta)}$	α and β are the shape parameters Γ is the gamma function
Bradford	$\frac{\alpha}{\log(1+\alpha)(1+\alpha x)}$	α is the shape parameter
Chi	$\frac{1}{2^{\alpha/2-1}\Gamma(\frac{\alpha}{2})} x^{\alpha-1} e^{-\frac{x^2}{2}}$	α is the degrees of freedom<br>Γ is the gamma function
Chi-squared	$\frac{1}{2^{\alpha/2}\Gamma(\frac{\alpha}{2})} x^{\alpha/2-1} e^{-\frac{x}{2}}$	α is the degrees of freedom<br>Γ is the gamma function
Double gamma	$\frac{\alpha}{2\Gamma(\alpha)}\lvert x\rvert^{\alpha-1}e^{-\lvert x\rvert}$	α is the shape parameter<br>Γ is the gamma function
Double Weibull	$\frac{\alpha}{2}\lvert x\rvert^{\alpha-1}e^{-\lvert x\rvert^{\alpha}}$	α is the shape parameter
Exponential normal	$\frac{1}{2\alpha} e^{\frac{1}{2\alpha^2}-\frac{x}{\alpha}} erfc(-\frac{x-\frac{1}{\alpha}}{\sqrt{2}})$	α is the shape parameter
Exponential Weibull	$\alpha\beta(1-e^{-x^{\beta}})^{\alpha-1}e^{-x^{\beta}}x^{\beta-1}$	α and β are the shape parameters
Exponential power	$\alpha x^{\alpha-1}e^{1+x^{\alpha}-e^{x^{\alpha}}}$	α is the shape parameter
Gamma		
Generalized logistic	$\alpha\frac{e^{-x}}{(1+e^{-x})^{\alpha+1}}$	α is the shape parameter
Generalized Pareto	$(1+\alpha x)^{-1-\frac{1}{\alpha}}$	α is the shape parameter
Generalized normal	$\frac{\alpha}{2\Gamma(1/\alpha)}e^{-\lvert x\rvert^{\alpha}}$	α is the shape parameter<br>Γ is the gamma function
Generalized exponential	$(\alpha+\beta(1-e^{\gamma x}))e^{-\alpha x-\beta x+\frac{\beta}{\gamma}(1-e^{-\gamma x})}$	α, β, γ are the shape parameters

Generalized gamma	$\frac{\lvert\beta\rvert x^{\alpha\beta-1}}{\Gamma(\alpha)}e^{-x^{\beta}}$	α and β are the shape parameters
Half-logistic	$\frac{2e^{-x}}{(1+e^{-x})^2}$	
Half-normal	$\sqrt{2/\pi}e^{\frac{-x^2}{2}}$	
Upped half of the generalized normal	$\frac{\alpha}{\Gamma(1/\alpha)}e^{-\lvert x\rvert^{\alpha}}$	α is the shape parameter
Inverse-gamma	$\frac{x^{-\alpha-1}}{\Gamma(\alpha)}e^{-\frac{1}{x}}$	α is the shape parameter
Inverse-normal	$\frac{1}{\sqrt{2\pi x^3}}e^{-\frac{(x-\alpha)^2}{2x\alpha^2}}$	α is the shape parameter
Inverse-Weibull	$\alpha x^{-\alpha-1}e^{-x^{-\alpha}}$	α is the shape parameter
Laplace	$\frac{1}{2}e^{-\lvert x\rvert}$	
Log-gamma	$\frac{e^{\alpha x-e^{x}}}{\Gamma(\alpha)}$	α is the shape parameter<br>Γ is the gamma function
Logistic	$\frac{e^{-x}}{(1+e^{-x})^2}$	
Log-Laplace	$\frac{\alpha}{2}x^{\alpha-1} \quad 0 < x < 1$<br>$\frac{\alpha}{2}x^{-\alpha-1} \quad x \geq 1$	α is the shape parameter
Log-normal	$\frac{1}{\alpha x\sqrt{2\pi}}e^{-\frac{(\log x)^2}{2\alpha^2}}$	α is the shape parameter
Maxwell	$\sqrt{2/\pi}x^2e^{\frac{-x^2}{2}}$	
Normal	$\frac{1}{\sqrt{2\pi}}e^{-\frac{x^2}{2}}$	
Pareto	$\frac{\alpha}{x^{\alpha+1}}$	α is the shape parameter
Power-law	$\alpha x^{\alpha-1}$	α is the shape parameter
Power Log-normal	$\frac{\alpha}{x\beta}\phi(\frac{\log x}{\beta})(\Phi\left(-\frac{\log x}{\beta}\right))^{\alpha-1}$	α and β are the shape parameters<br>ϕ is the normal PDF<br>Φ is the normal CDF
Power normal	$\alpha\phi(\mathrm{x})(\Phi(-\mathrm{x}))^{\alpha-1}$	α is the shape parameter<br>ϕ is the normal PDF<br>Φ is the normal CDF
Uniform	1	
Weibull maximum	$\alpha(-x)^{\alpha-1}e^{-(-x)^{\alpha}}$	α is the shape parameter
Weibull minimum	$\alpha x^{\alpha-1}e^{-x^{\alpha}}$	α is the shape parameter

Gamma function: $\Gamma(\alpha) = \int_0^{\infty} x^{\alpha-1}e^{-x}dx = (\alpha-1)!$

FIGURES

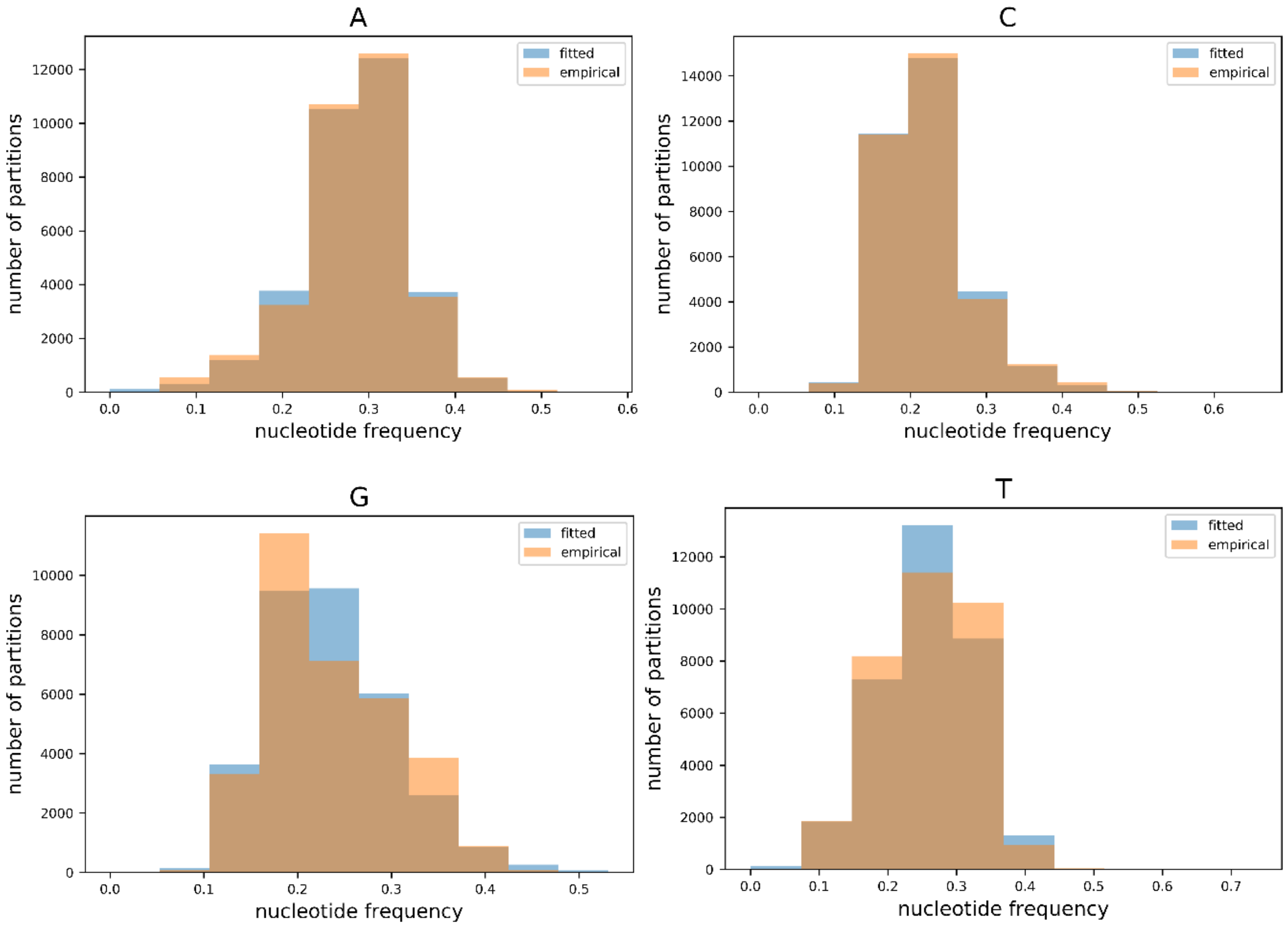

FIGURE A.1. distribution of the nucleotide frequencies. The empirical nucleotide frequencies for each single partition were estimated using IQ-TREE (orange) and the Fitted distribution (blue) were sampled from the best-fit distribution with the same number of partitions.

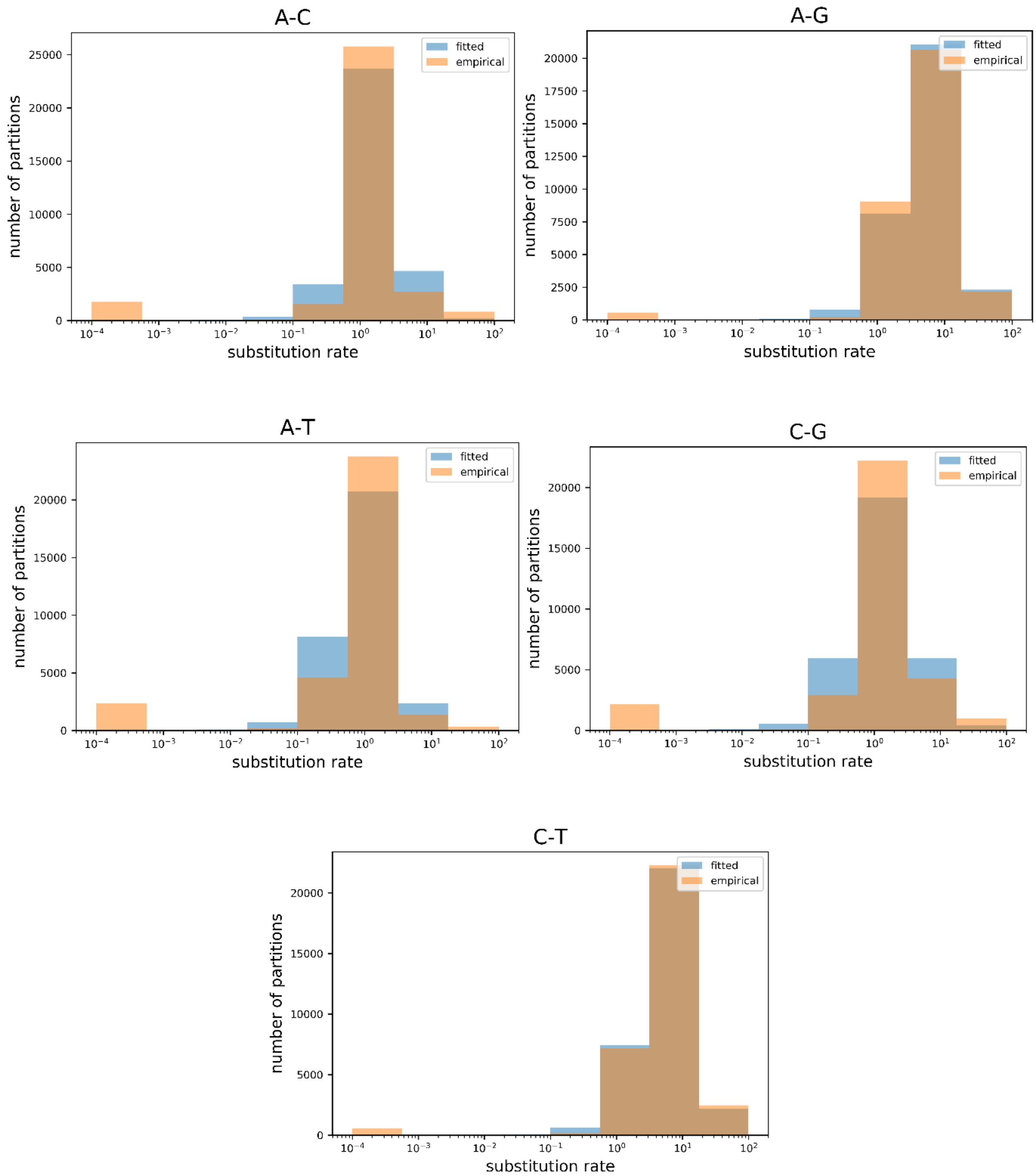

FIGURE A.2. distribution of the GTR parameters. The best-fit substitution rate matrix for each single partition was estimated using IQ-TREE (orange) and the Fitted distribution (blue) were sampled from the best-fit distribution with the same number of partitions.

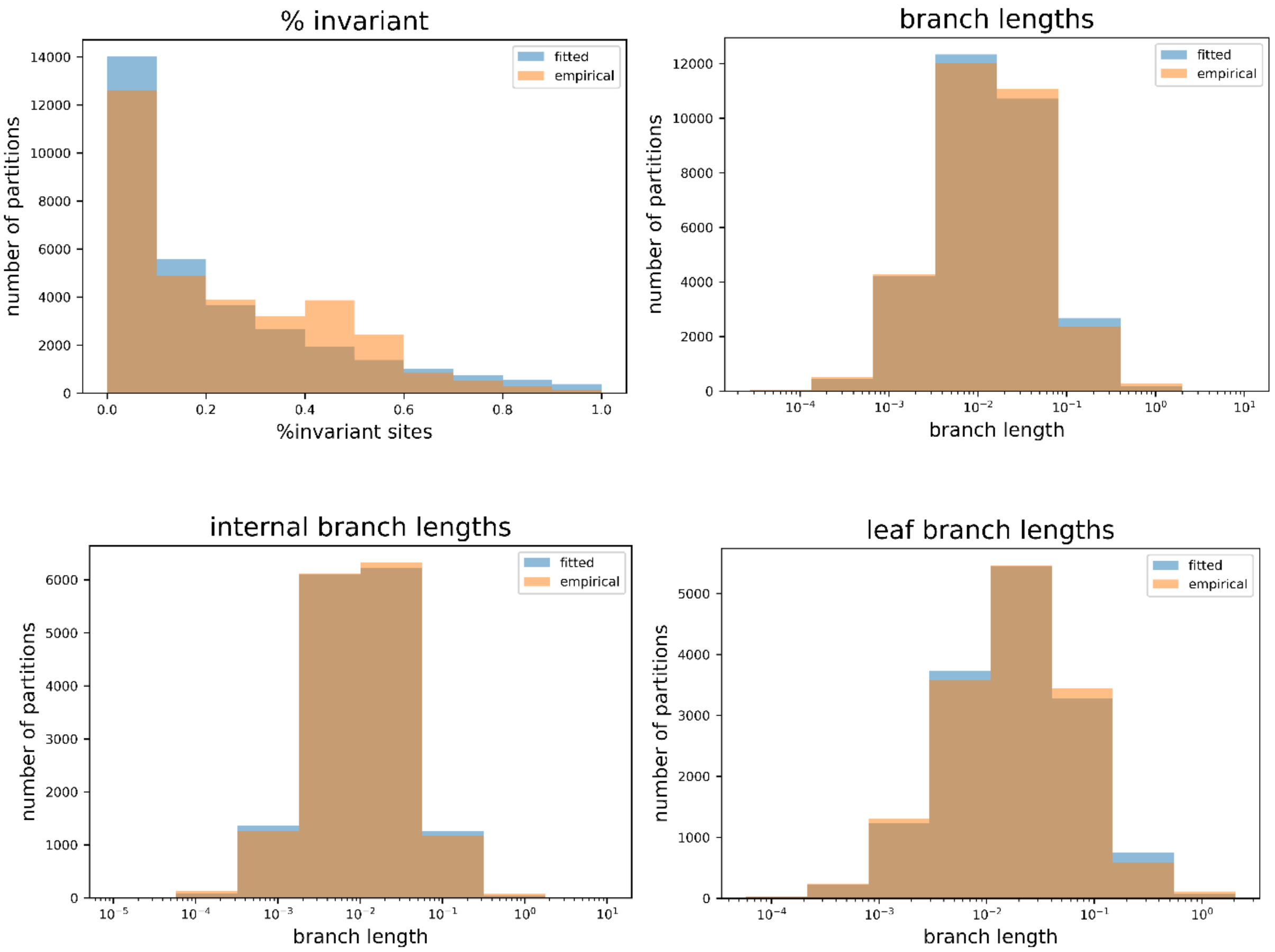

FIGURE A.3. The distribution of branch lengths and proportion of invariant sites

a)

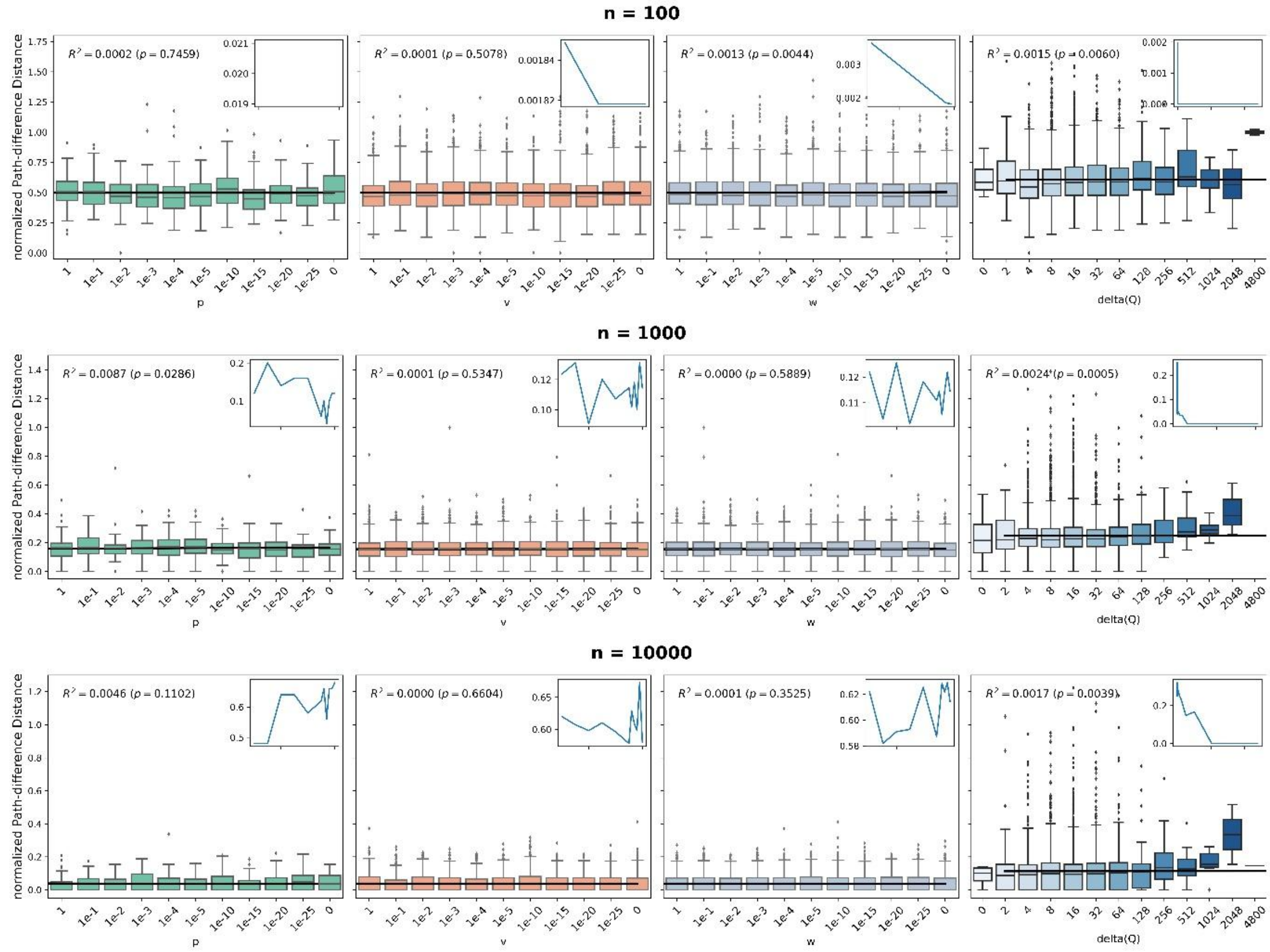
n = 100
normalized Path-difference Distance
$R^2 = 0.0002$ ($p = 0.7459$)
$R^2 = 0.0001$ ($p = 0.5078$)
$R^2 = 0.0013$ ($p = 0.0044$)
$R^2 = 0.0015$ ($p = 0.0060$)
p
v
w
delta(Q)
n = 1000
$R^2 = 0.0087$ ($p = 0.0286$)
$R^2 = 0.0001$ ($p = 0.5347$)
$R^2 = 0.0000$ ($p = 0.5889$)
$R^2 = 0.0024$ ($p = 0.0005$)
n = 10000
$R^2 = 0.0046$ ($p = 0.1102$)
$R^2 = 0.0000$ ($p = 0.6604$)
$R^2 = 0.0001$ ($p = 0.3525$)
$R^2 = 0.0017$ ($p = 0.0039$)

b)

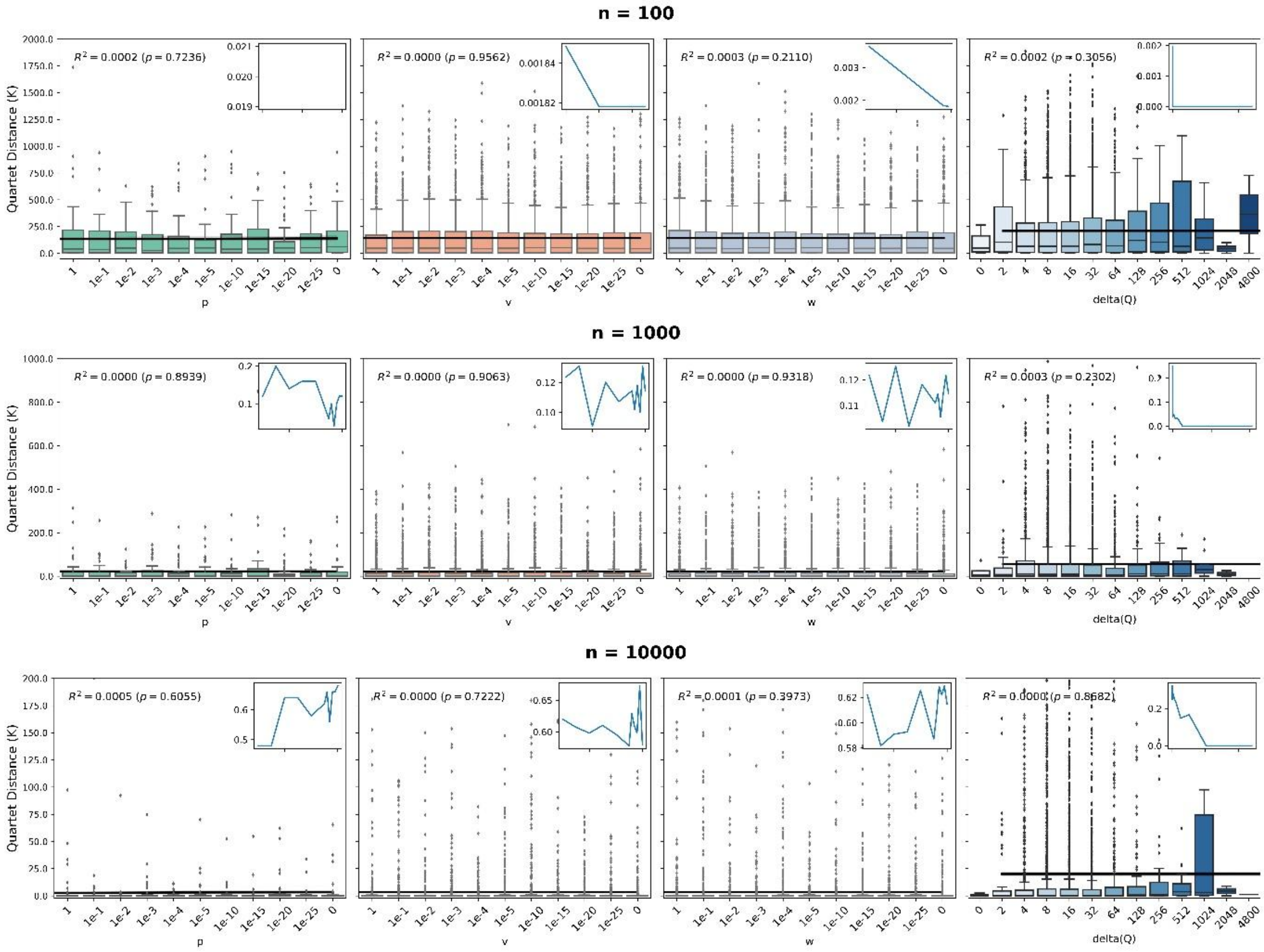

FIGURE A.4. (a) Normalized Path-difference and (b) Quartet metrics between the estimated tree topology and the original tree topology as a function of the inheritance weight (ν, ω ,ρ) and the distance between the two matrices. The small plots show the proportion of datasets in which the distance between the estimated topology and the original topology equals to zero as a function of the inheritance weight and the distance between the two matrices.

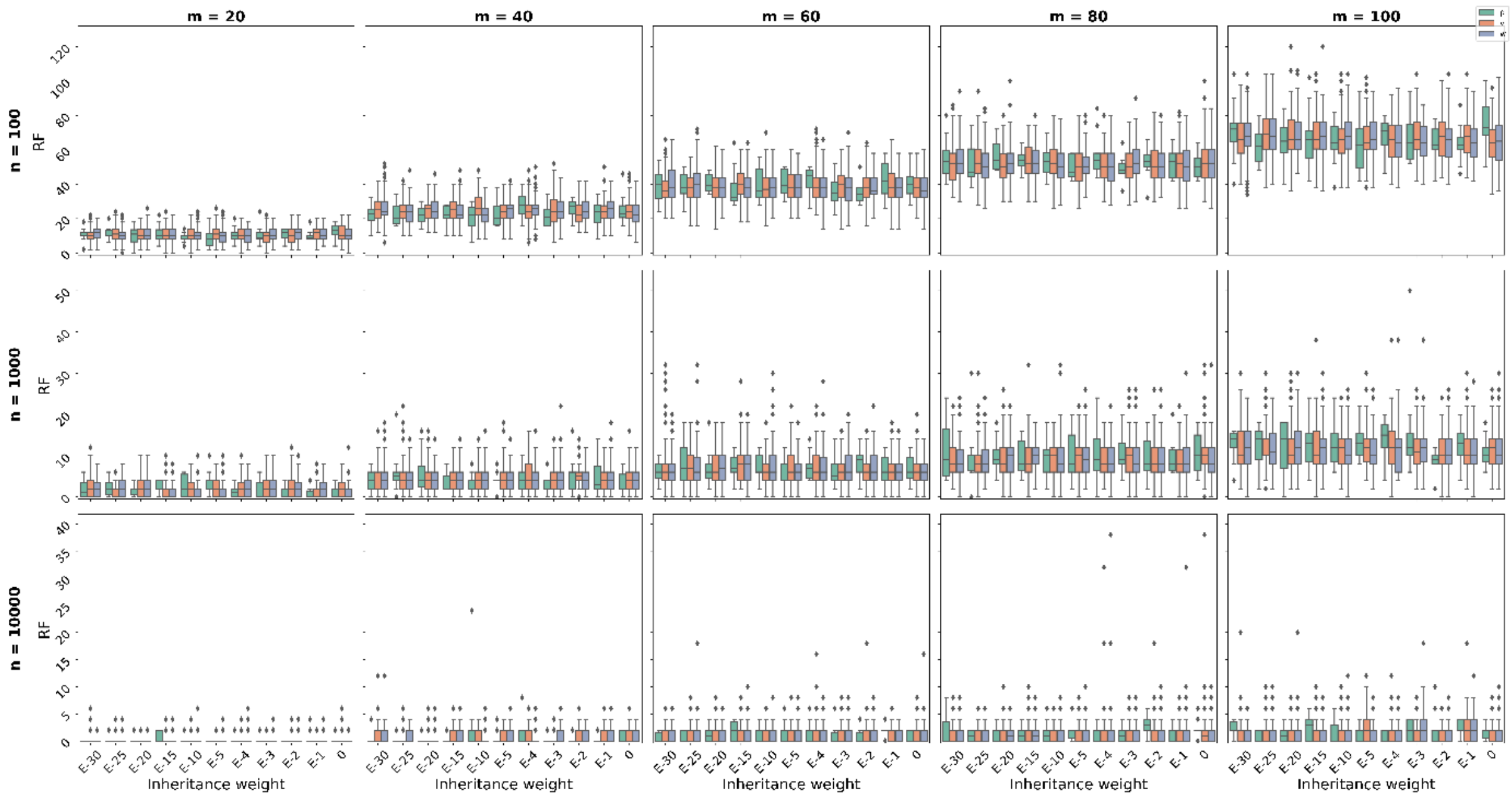

FIGURE A.5. The Robinson-Foulds metric as a function of the inheritance weight for each number of taxa (m) and number of site (n).

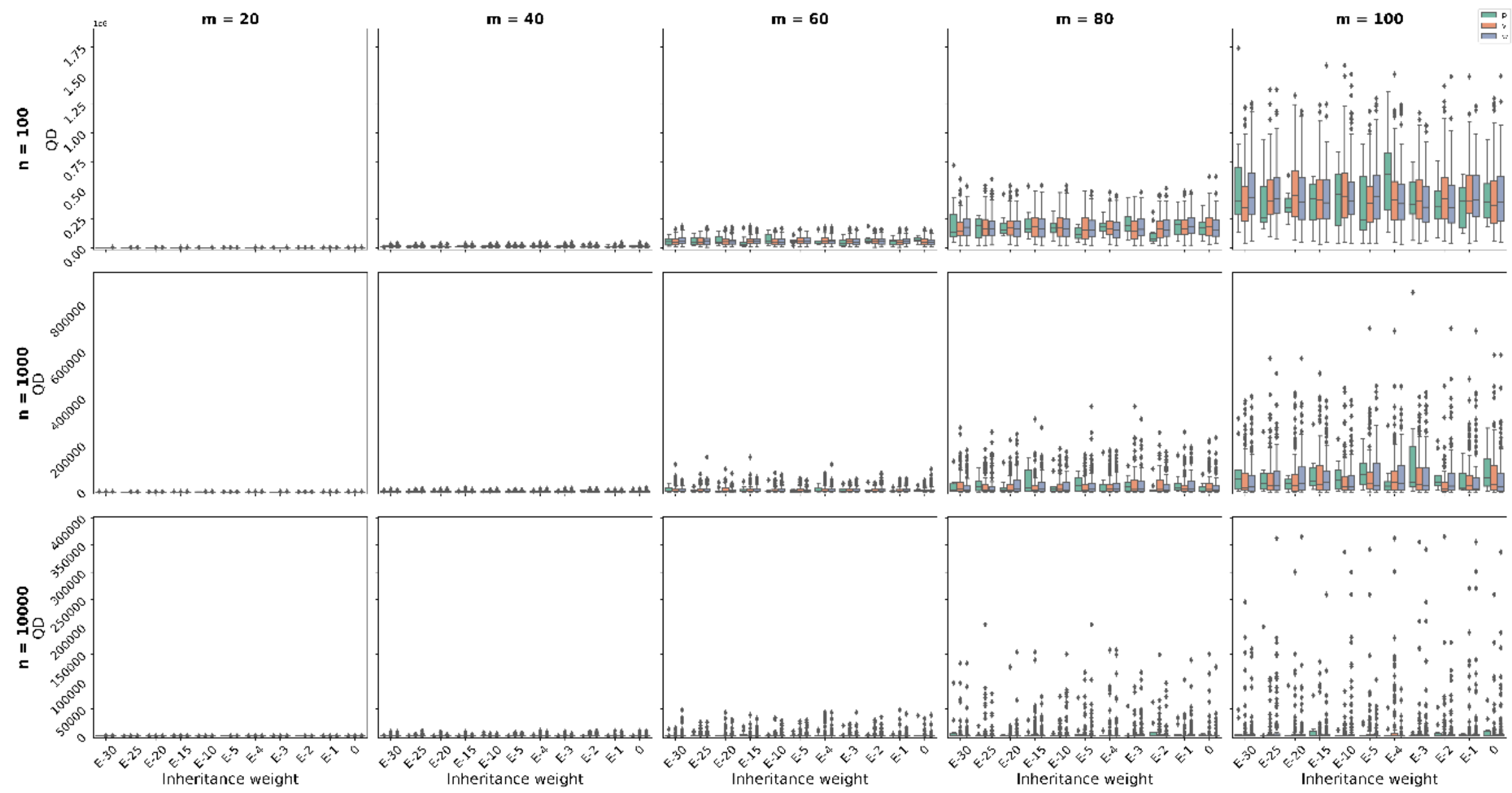

FIGURE A.6. The Quartet distance as a function of the inheritance weight for each number of taxa (m) and number of site (n).

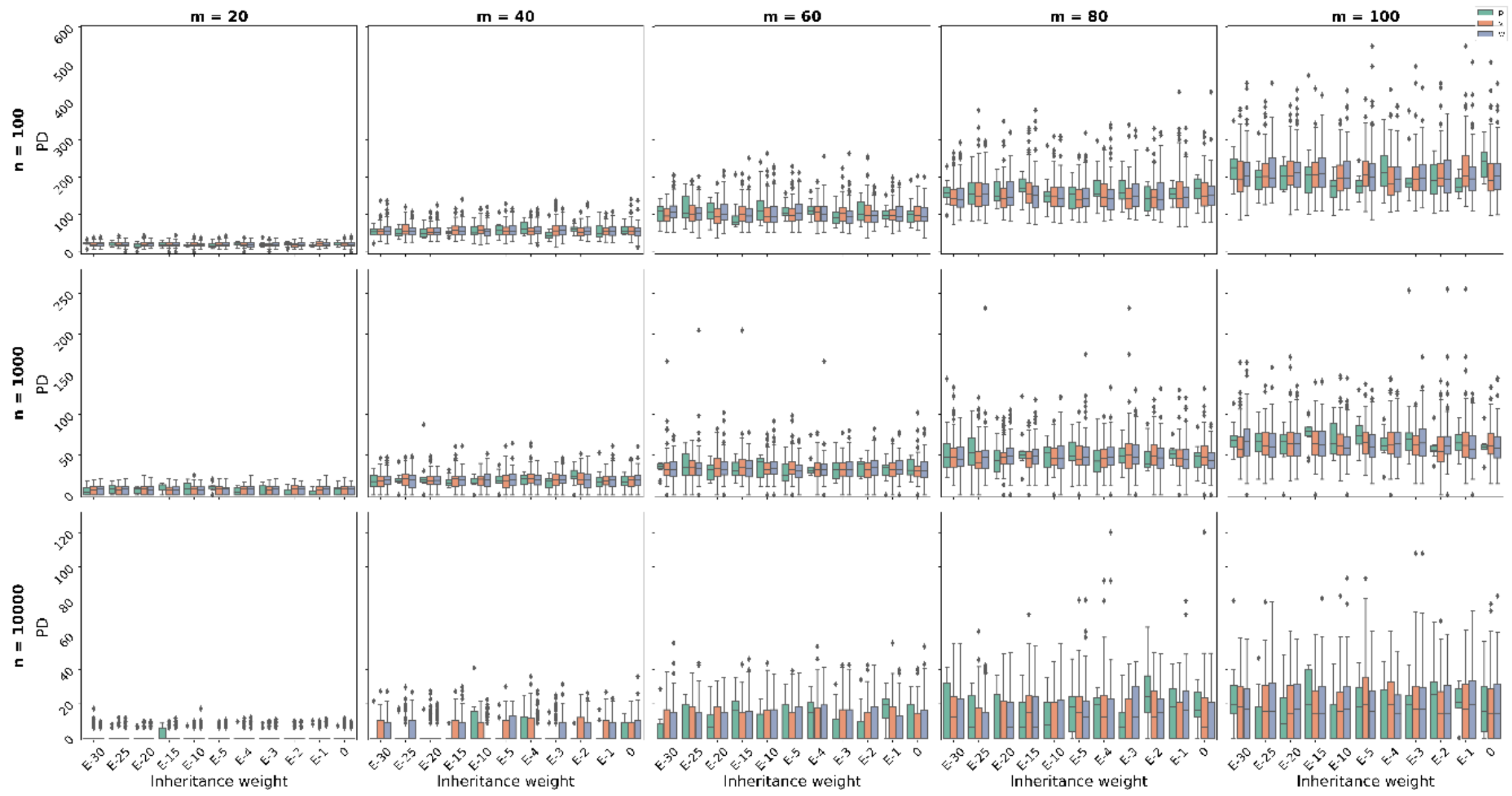

FIGURE A.7. The Path-Difference distance as a function of the inheritance weight for each number of taxa (m) and number of site (n).

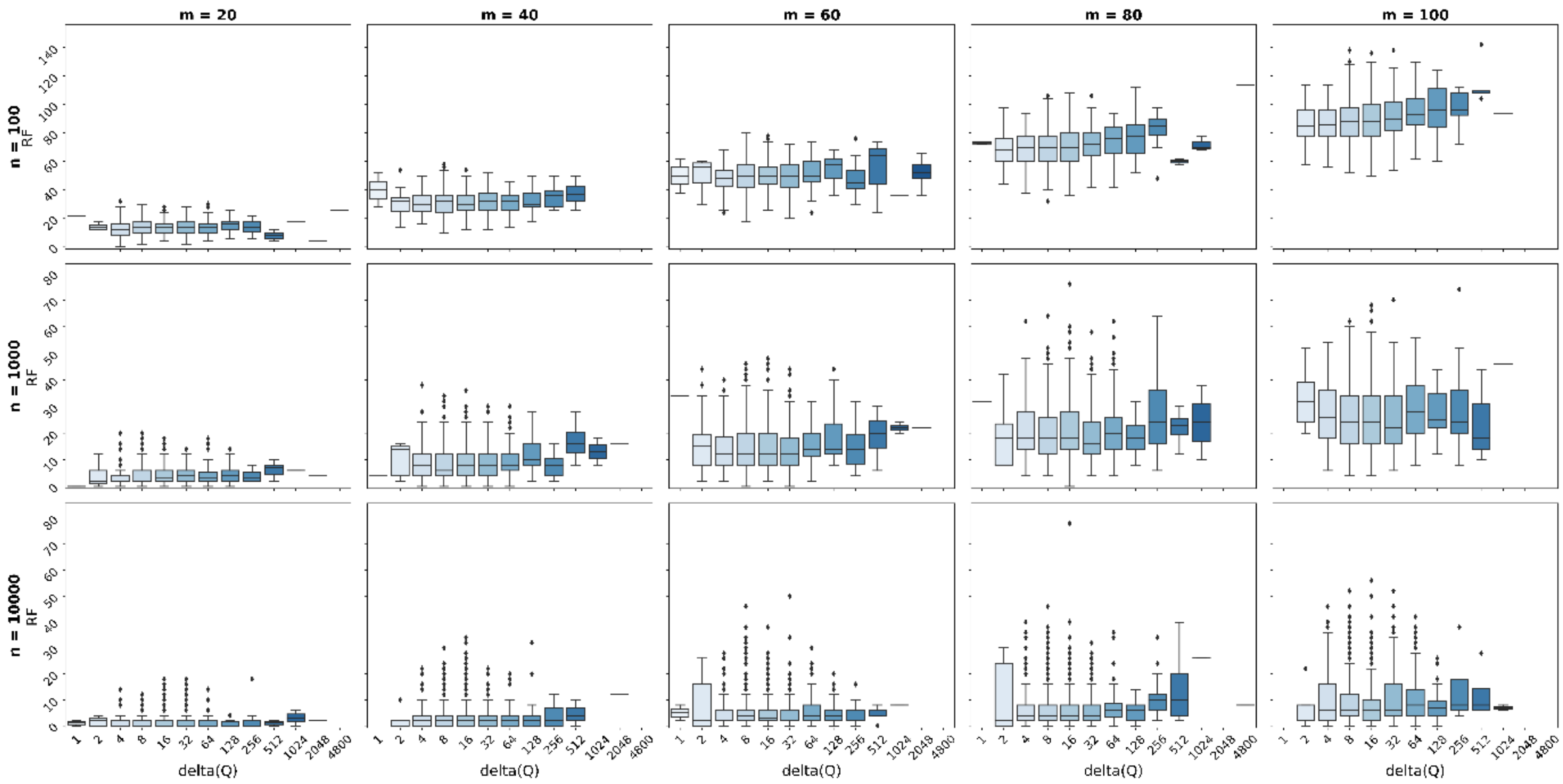

FIGURE A.8. The Robinson-Foulds metric as a function of the maximum Euclidian distance between the two matrices for each number of taxa (m) and number of site (n).

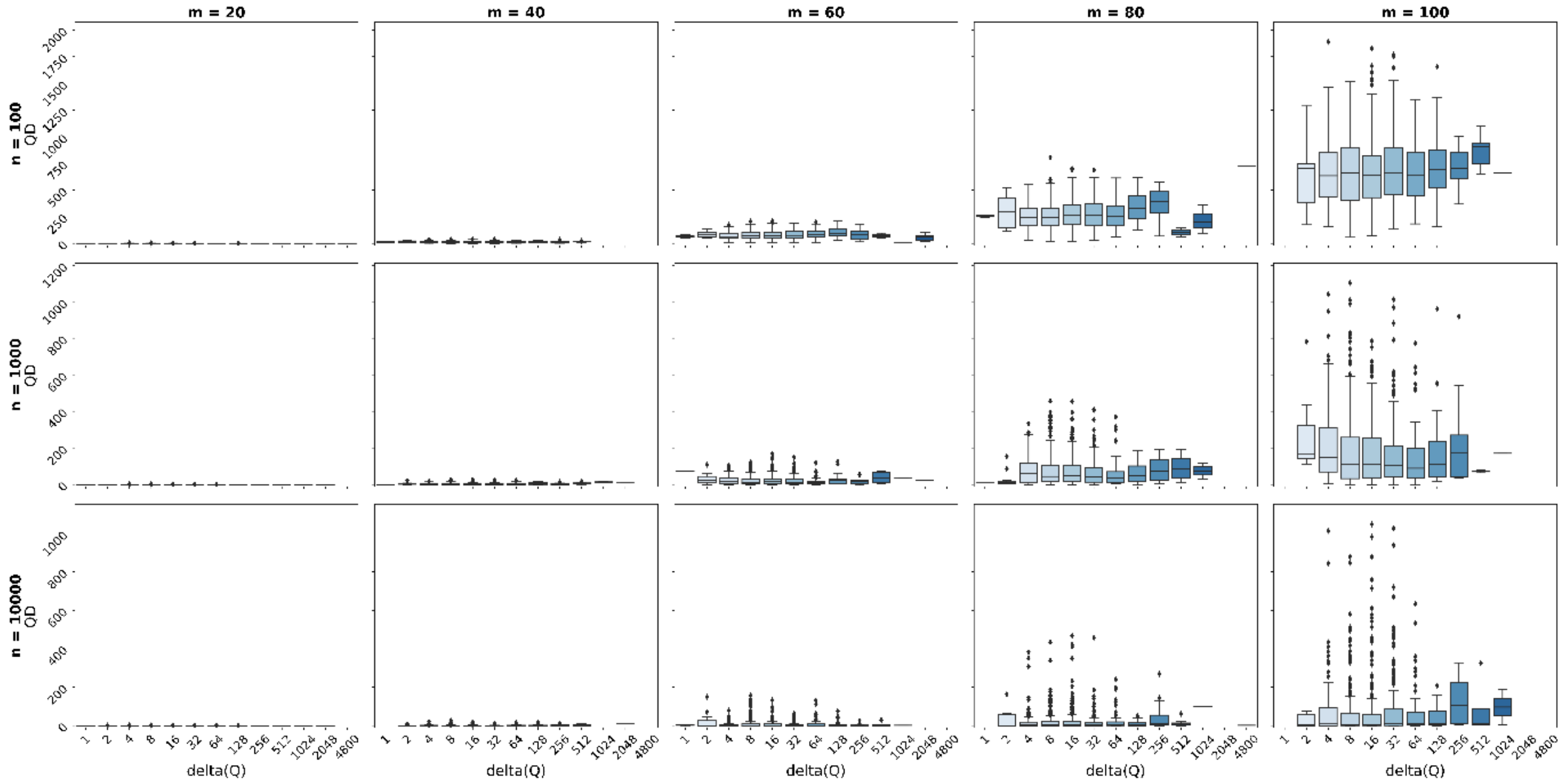

FIGURE A.9. The Quartet distance as a function of the maximum Euclidian distance between the two matrices for each number of taxa (m) and number of site (n).

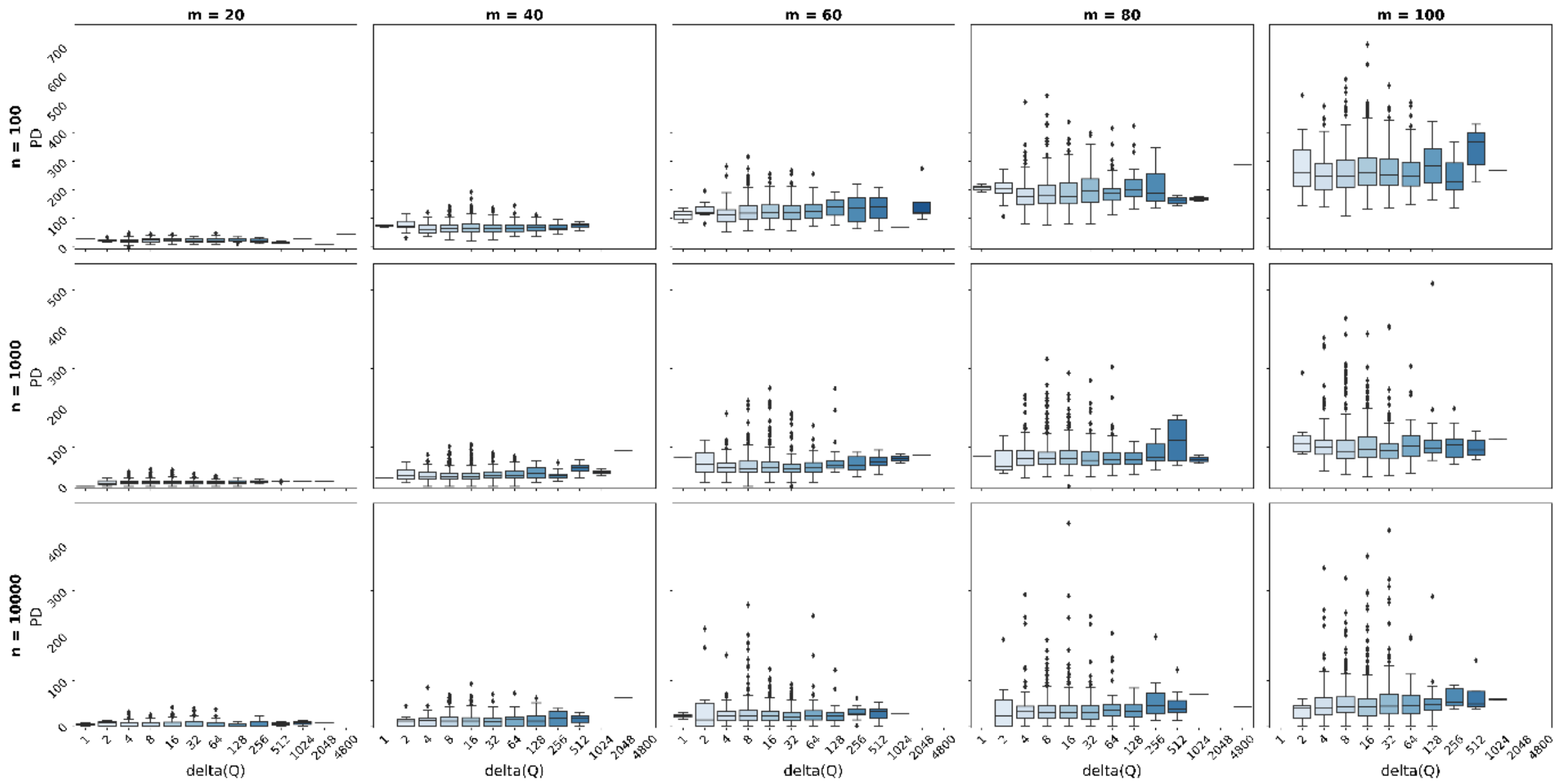

FIGURE A.10. The Path-Difference distance as a function of the maximum Euclidian distance between the two matrices for each number of taxa (m) and number of site (n).

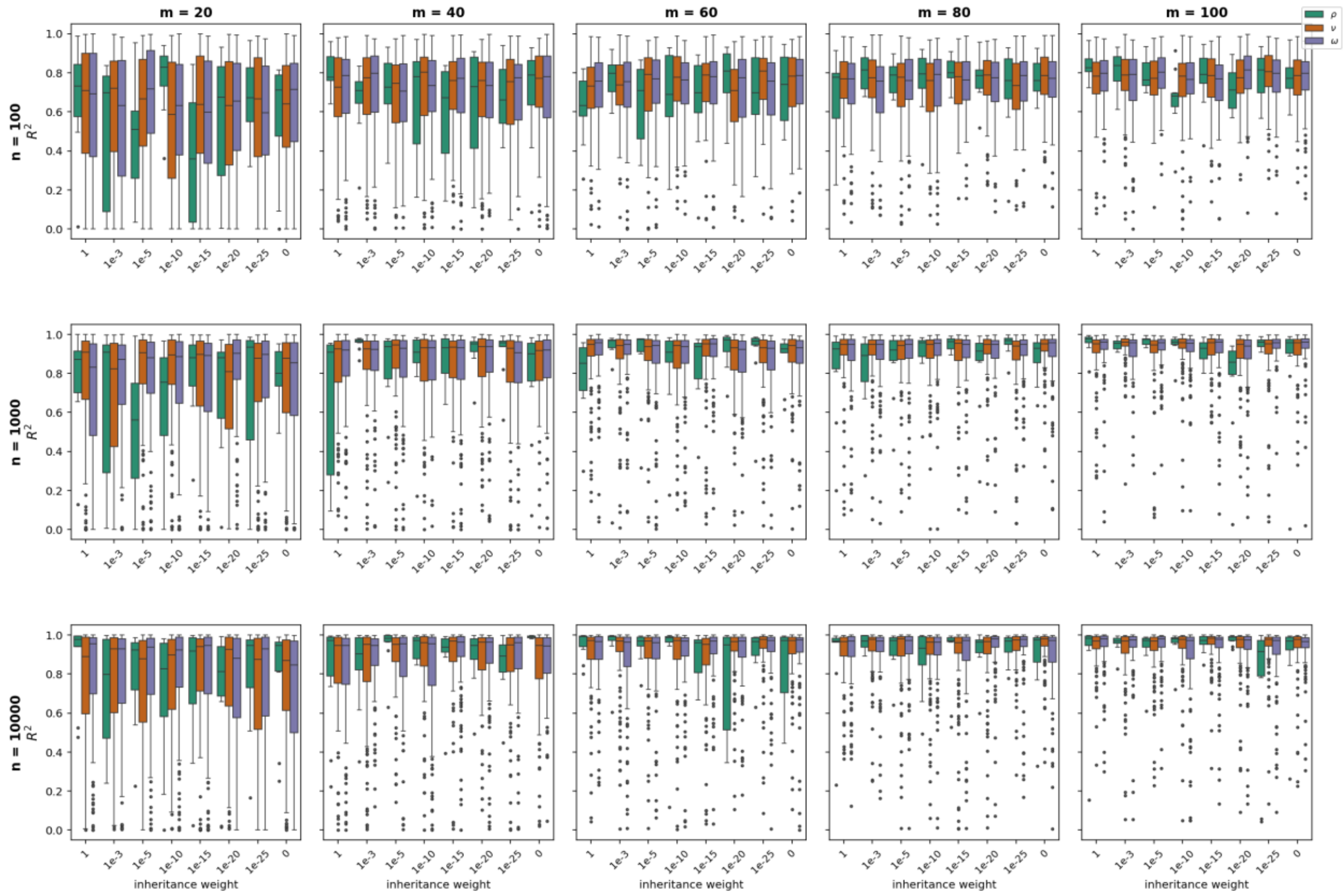

FIGURE A.11. The accuracy of branch length estimation (R^2) as a function of the inheritance weight of the base frequencies (ν), the substitution model (ρ), the inheritance weight of the substitution rates (ω) for each number of taxa (m) and number of site (n).

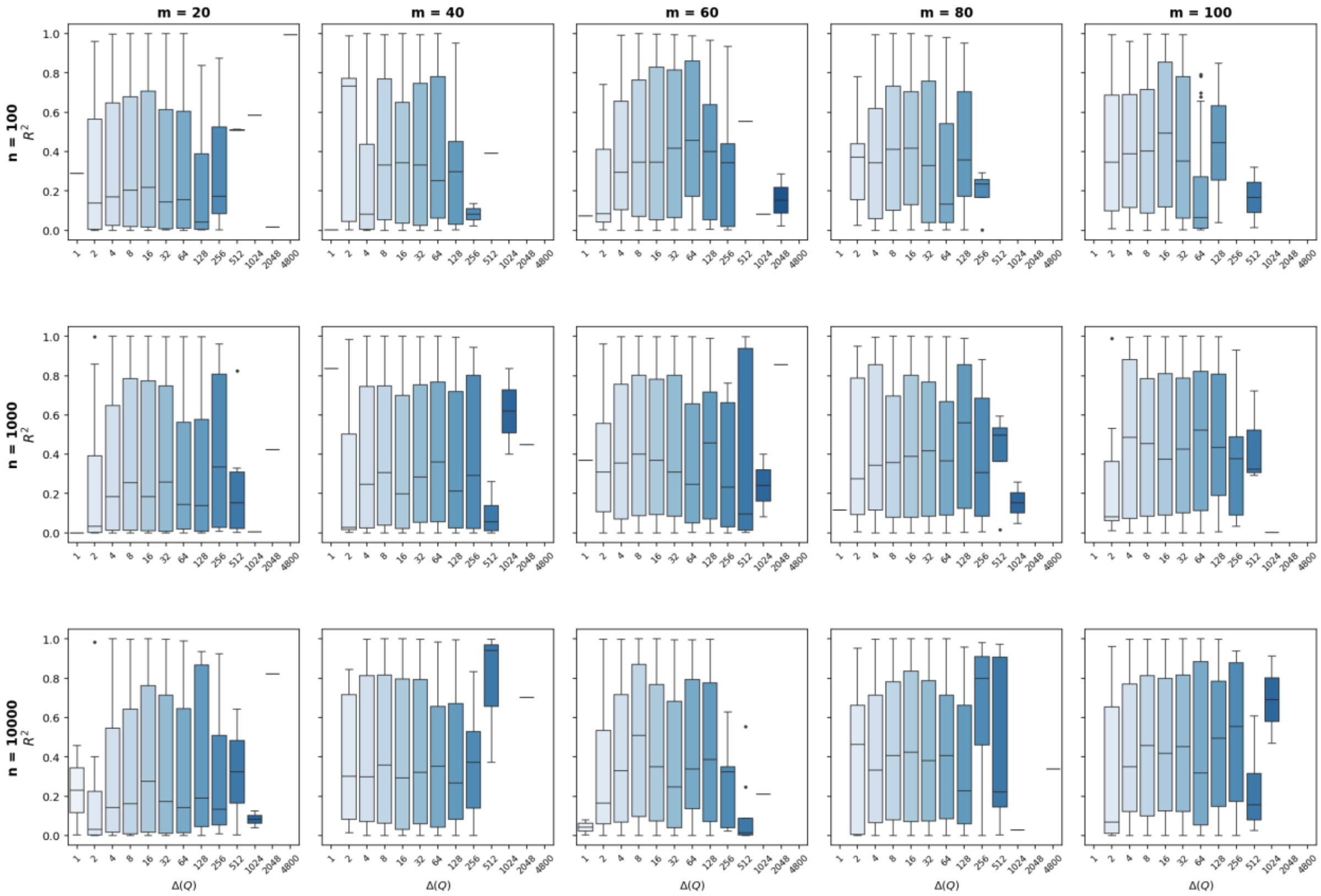

FIGURE A.12. The accuracy of branch length estimation (R^2) as a function of the inheritance weight of the Euclidean distance between the two matrices for each number of taxa (m) and number of sites (n).

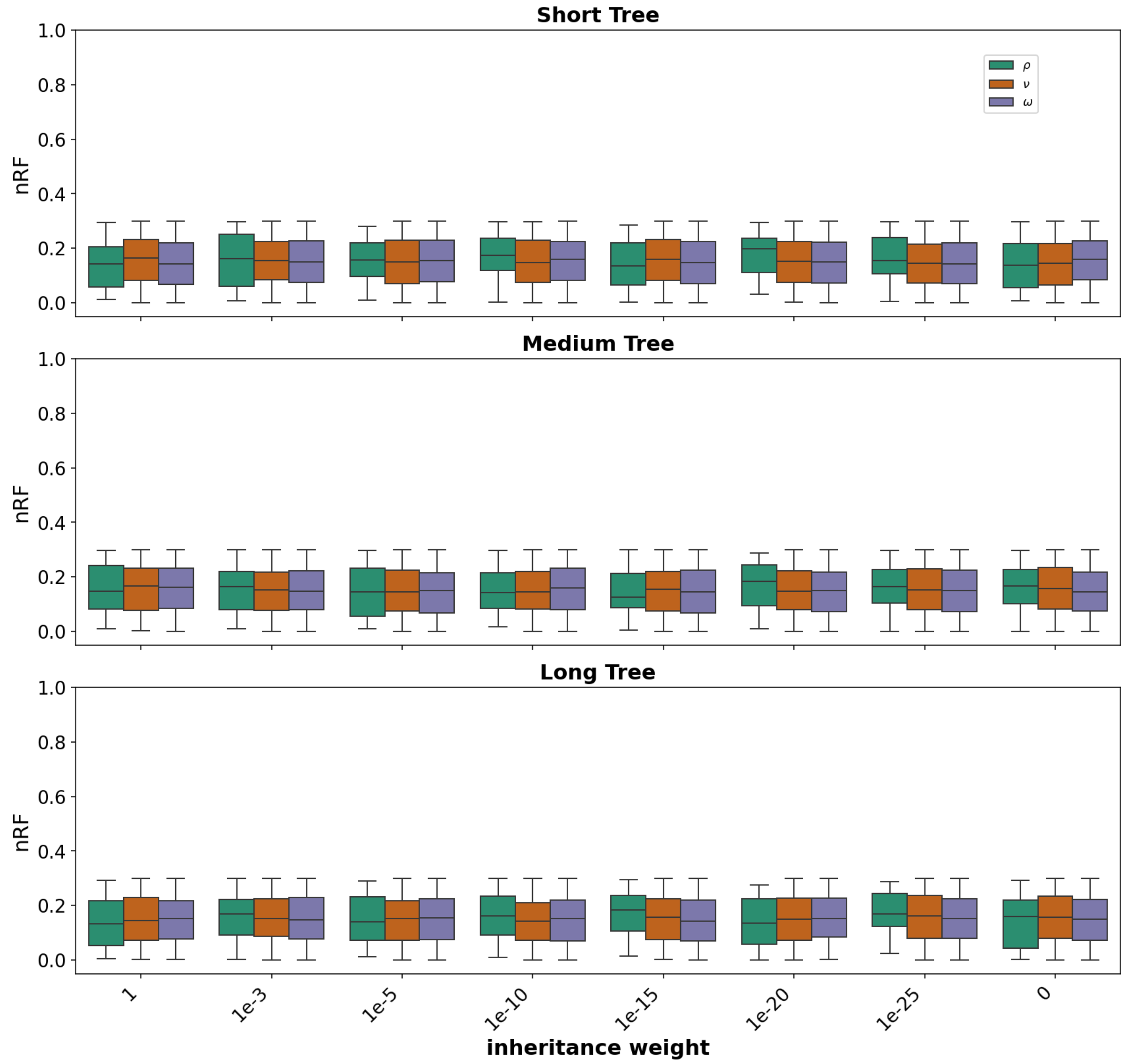

FIGURE A.13. The normalized Robinson-Foulds metric as a function of the inheritance weight of the base frequencies (ν), the substitution model (ρ), the inheritance weight of the substitution rates (ω) for each category of tree length, based on the tertiles of the total tree length. Short Tree: trees with total length between 2.17 and 5.52. Medium Tree: trees with total length between 5.52 and 9.95. Long Tree: trees with total length between 9.95 and 15.39.

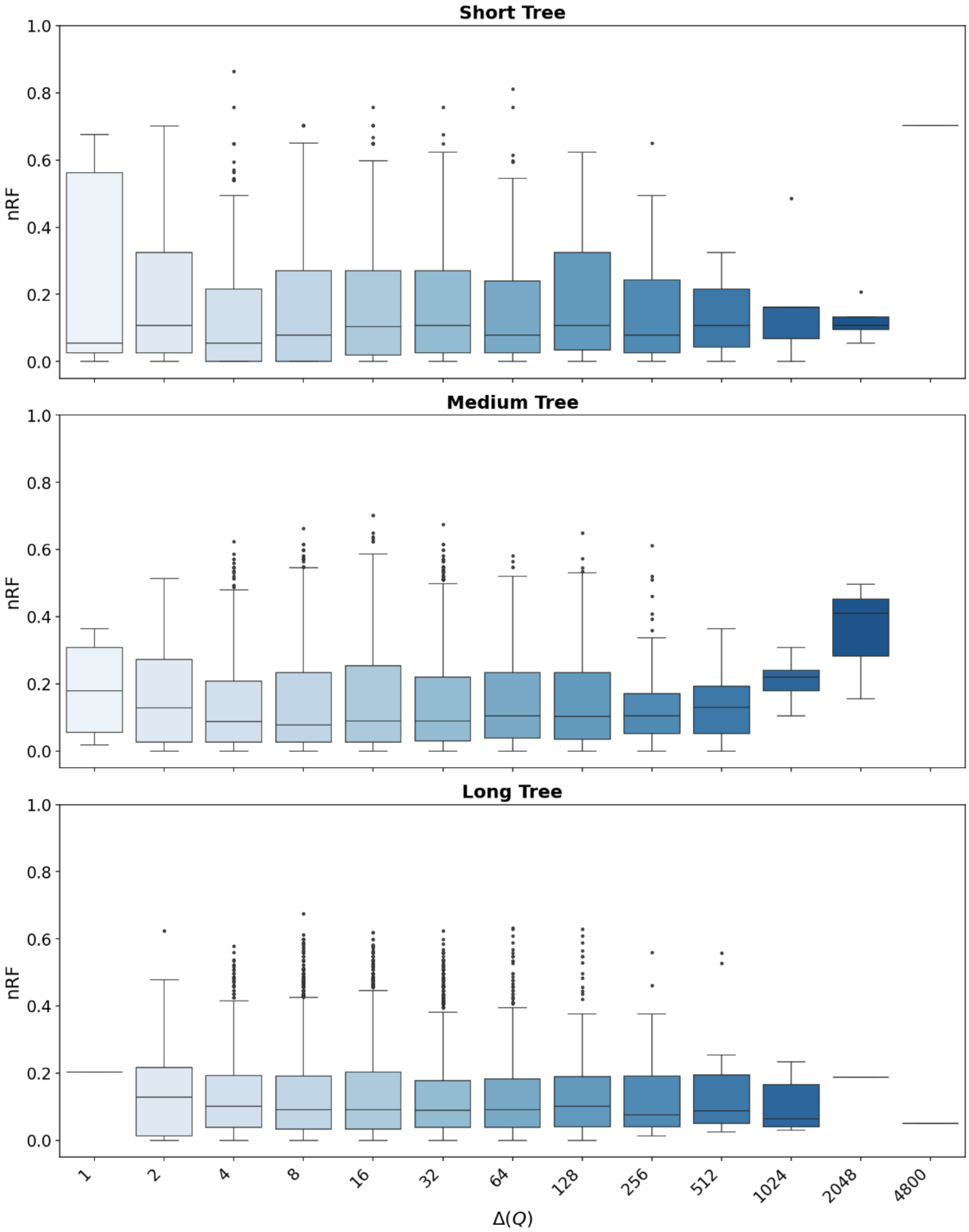

FIGURE A.14. The normalized Robinson-Foulds as a function of the inheritance weight of the Euclidean distance between the two matrices for each category of tree length, based on the tertiles of the total tree length. Short Tree: trees with total length between 2.38 and 9.06. Medium Tree: trees with total length between 9.06 and 16.86. Long Tree: trees with total length between 16.86 and 37.15.

References

Anderson FE, Bergman A, Cheng SH, Pankey MS, Valinassab T. 2013. Data from: Lights out: the evolution of bacterial bioluminescence in Loliginidae. In: Dryad Data Repository.

Anderson FE, Bergman A, Cheng SH, Pankey MS, Valinassab T. 2014. Lights out: the evolution of bacterial bioluminescence in Loliginidae. Hydrobiologia 725:189-203.

Ballesteros JA, Sharma PP. 2019a. A Critical Appraisal of the Placement of Xiphosura (Chelicerata) with Account of Known Sources of Phylogenetic Error. Syst. Biol.

Ballesteros JA, Sharma PP. 2019b. Data from: A critical appraisal of the placement of Xiphosura (Chelicerata) with account of known sources of phylogenetic error. In: Dryad.

Becker EA, Yao AI, Seitzer PM, Kind T, Wang T, Eigenheer R, Shao KS, Yarov-Yarovoy V, Facciotti MT. 2016. A Large and Phylogenetically Diverse Class of Type 1 Opsins Lacking a Canonical Retinal Binding Site. PLoS One 11:e0156543.

Becker EA, Yao AI, Seitzer PM, Kind T, Wang T, Eigenheer R, Shao KSY, Yarov-Yarovoy V, Facciotti MT. 2017. Data from: A large and phylogenetically diverse class of type 1 opsins lacking a canonical retinal binding site. In: Dryad.

Bergsten J, Nilsson AN, Ronquist F. 2013a. Bayesian tests of topology hypotheses with an example from diving beetles. Syst. Biol. 62:660-673.

Bergsten J, Nilsson AN, Ronquist F. 2013b. Data from: Bayesian tests of topology hypotheses with an example from diving beetles. In: Dryad Data Repository.

Borowiec ML, Lee EK, Chiu JC, Plachetzki DC. 2016. Data from: Extracting phylogenetic signal and accounting for bias in whole-genome data sets supports the Ctenophora as sister to remaining Metazoa. In: Dryad Digital Repository.

Borowiec ML, Lee EK, Chiu JC, Plachetzki DC. 2015. Extracting phylogenetic signal and accounting for bias in whole-genome data sets supports the Ctenophora as sister to remaining Metazoa. BMC Genomics 16:987.

Branstetter MG, Danforth BN, Pitts JP, Faircloth BC, Ward PS, Buffington ML, Gates MW, Kula RR, Brady SG. 2017a. Data from: Phylogenomic insights into the evolution of stinging wasps and the origins of ants and bees. In: Dryad Digital Repository.

Branstetter MG, Danforth BN, Pitts JP, Faircloth BC, Ward PS, Buffington ML, Gates MW, Kula RR, Brady SG. 2017b. Phylogenomic Insights into the Evolution of Stinging Wasps and the Origins of Ants and Bees. Curr. Biol. 27:1019-1025.

Broughton RE, Betancur RR, Li C, Arratia G, Orti G. 2013a. Data from: Multi-locus phylogenetic analysis reveals the pattern and tempo of bony fish evolution. In: Dryad Data Repository.

Broughton RE, Betancur RR, Li C, Arratia G, Orti G. 2013b. Multi-locus phylogenetic analysis reveals the pattern and tempo of bony fish evolution. PLoS Curr 5.

Brown RM, Siler CD, Das I, Min PY. 2012a. Data from: Testing the phylogenetic affinities of Southeast Asia's rarest geckos: Flap-legged geckos (Luperosaurus), Flying geckos (Ptychozoon) and their relationship to the pan-Asian genus Gekko. In: Dryad Data Repository.

Brown RM, Siler CD, Das I, Min Y. 2012b. Testing the phylogenetic affinities of Southeast Asia's rarest geckos: Flap-legged geckos (Luperosaurus), Flying geckos (Ptychozoon) and their relationship to the pan-Asian genus Gekko. Mol Phylogenet Evol 63:915-921.

Cannon JT, Vellutini BC, Smith J, 3rd, Ronquist F, Jondelius U, Hejnol A. 2016a. Xenacoelomorpha is the sister group to Nephrozoa. Nature 530:89-93.

Cannon JT, Vellutini BC, Smith J, Ronquist F, Jondelius U, Hejnol A. 2016b. Data from: Xenacoelomorpha is the sister group to Nephrozoa. In: Dryad Data Repository.

Chen M-Y, Liang D, Zhang P. 2015a. Data from: Selecting question-specific genes to reduce incongruence in phylogenomics: a case study of jawed vertebrate backbone phylogeny. In: Dryad.

Chen MY, Liang D, Zhang P. 2015b. Selecting Question-Specific Genes to Reduce Incongruence in Phylogenomics: A Case Study of Jawed Vertebrate Backbone Phylogeny. Syst. Biol. 64:1104-1120.

Cognato AI, Vogler AP. 2001a. Data from: Exploring data interaction and nucleotide alignment in a multiple gene analysis of Ips (Coleoptera: Scolytinae). In: Dryad Data Repository.

Cognato AI, Vogler AP. 2001b. Exploring data interaction and nucleotide alignment in a multiple gene analysis of Ips (Coleoptera: Scolytinae). Syst. Biol. 50:758-780.

Crawford NG, Faircloth BC, McCormack JE, Brumfield RT, Winker K, Glenn TC. 2012a. Data from: More than 1000 ultraconserved elements provide evidence that turtles are the sister group of archosaurs. In: Dryad Digital Repository.

Crawford NG, Faircloth BC, McCormack JE, Brumfield RT, Winker K, Glenn TC. 2012b. More than 1000 ultraconserved elements provide evidence that turtles are the sister group of archosaurs. Biol. Lett. 8:783-786.

Day JJ, Peart CR, Brown KJ, Friel JP, Bills R, Moritz T. 2013a. Continental diversification of an African catfish radiation (Mochokidae: Synodontis). Syst. Biol. 62:351-365.

Day JJ, Peart CR, Brown KJ, Friel JP, Bills R, Moritz T. 2013b. Data from: Continental diversification of an African catfish radiation (Mochokidae: Synodontis). In: Dryad Data Repository.

Devitt TJ, Cameron Devitt SE, Hollingsworth BD, McGuire JA, Moritz C. 2013. Data from: Montane refugia predict population genetic structure in the Large-blotched Ensatina salamander. In: Dryad Data Repository.

Devitt TJ, Devitt SE, Hollingsworth BD, McGuire JA, Moritz C. 2013. Montane refugia predict population genetic structure in the Large-blotched Ensatina salamander. Mol. Ecol. 22:1650-1665.

Dornburg A, Moore JA, Webster R, Warren DL, Brandley MC, Iglesias TL, Wainwright PC, Near TJ. 2012a. Data from: Molecular phylogenetics of squirrelfishes and soldierfishes (Teleostei:Beryciformes: Holocentridae): reconciling more than 100 years of taxonomic confusion. In: Dryad Data Repository.

Dornburg A, Moore JA, Webster R, Warren DL, Brandley MC, Iglesias TL, Wainwright PC, Near TJ. 2012b. Molecular phylogenetics of squirrelfishes and soldierfishes (Teleostei: Beryciformes: Holocentridae): reconciling more than 100 years of taxonomic confusion. Mol Phylogenet Evol 65:727-738.

Faircloth BC, Sorenson L, Santini F, Alfaro ME. 2013a. Data from: A phylogenomic perspective on the radiation of ray-finned fishes based upon targeted sequencing of ultraconserved elements (UCEs). In: Dryad Data Repository.

Faircloth BC, Sorenson L, Santini F, Alfaro ME. 2013b. A Phylogenomic Perspective on the Radiation of Ray-Finned Fishes Based upon Targeted Sequencing of Ultraconserved Elements (UCEs). PLoS One 8:e65923.

Fong JJ, Brown JM, Fujita MK, Boussau B. 2012a. Data from: A phylogenomic approach to vertebrate phylogeny supports a turtle-archosaur affinity and a possible paraphyletic Lissamphibia. In: Dryad Data Repository.

Fong JJ, Brown JM, Fujita MK, Boussau B. 2012b. A phylogenomic approach to vertebrate phylogeny supports a turtle-archosaur affinity and a possible paraphyletic lissamphibia. PLoS One 7:e48990.

Horn JW, Xi Z, Riina R, Peirson JA, Yang Y, Dorsey BL, Berry PE, Davis CC, Wurdack KJ. 2014a. Data from: Evolutionary bursts in Euphorbia (Euphorbiaceae) are linked with photosynthetic pathway. In: Dryad Data Repository.

Horn JW, Xi Z, Riina R, Peirson JA, Yang Y, Dorsey BL, Berry PE, Davis CC, Wurdack KJ. 2014b. Evolutionary bursts in Euphorbia (Euphorbiaceae) are linked with photosynthetic pathway. Evolution 68:3485-3504.

Irisarri I, Baurain D, Brinkmann H, Delsuc F, Sire J, Kupfer A, Petersen J, Jarek M, Meyer A, Vences M, et al. 2017a. Data from: Phylotranscriptomic consolidation of the jawed vertebrate timetree. In: Dryad Digital Repository.

Irisarri I, Baurain D, Brinkmann H, Delsuc F, Sire JY, Kupfer A, Petersen J, Jarek M, Meyer A, Vences M, et al. 2017b. Phylotranscriptomic consolidation of the jawed vertebrate timetree. Nat Ecol Evol 1:1370-1378.

Jarvis ED, Mirarab S, Aberer A, Houde P, Li C, Ho S, Faircloth BC, Nabholz B, Howard JT, Suh A, et al. 2014. Data from: Phylogenomic analyses data of the avian phylogenomics project. In: GigaScience Database.

Jarvis ED, Mirarab S, Aberer AJ, Li B, Houde P, Li C, Ho SY, Faircloth BC, Nabholz B, Howard JT, et al. 2015. Phylogenomic analyses data of the avian phylogenomics project. Gigascience 4:4.

Kawahara AY, Rubinoff D. 2013a. Convergent evolution of morphology and habitat use in the explosive Hawaiian fancy case caterpillar radiation. J. Evol. Biol. 26:1763-1773.

Kawahara AY, Rubinoff D. 2013b. Data from: Convergent evolution in the explosive Hawaiian Fancy Cased caterpillar radiation. In: Dryad Data Repository.

Lartillot N, Delsuc F. 2012a. Data from: Joint reconstruction of divergence times and life-history evolution in placental mammals using a phylogenetic covariance model. In: Dryad Data Repository.

Lartillot N, Delsuc F. 2012b. Joint reconstruction of divergence times and life-history evolution in placental mammals using a phylogenetic covariance model. Evolution 66:1773-1787.

Leache AD, Chavez AS, Jones LN, Grummer JA, Gottscho AD, Linkem CW. 2015. Phylogenomics of phrynosomatid lizards: conflicting signals from sequence capture versus restriction site associated DNA sequencing. Genome Biol Evol 7:706-719.

Leaché AD, Chavez AS, Jones LN, Grummer JA, Gottscho AD, Linkem CW. 2015. Data from: Phylogenomics of phrynosomatid lizards: conflicting signals from sequence capture versus restriction site associated DNA sequencing. In: Dryad.

Looney BP, Ryberg M, Hampe F, Sanchez-Garcia M, Matheny PB. 2016. Into and out of the tropics: global diversification patterns in a hyperdiverse clade of ectomycorrhizal fungi. Mol. Ecol. 25:630-647.

Looney BP, Ryberg M, Hampe F, Sánchez-García M, Matheny PB. 2015. Data from: Into and out of the tropics: global diversification patterns in a hyper-diverse clade of ectomycorrhizal fungi. In: Dryad.

McCormack JE, Harvey MG, Faircloth BC, Crawford NG, Glenn TC, Brumfield RT. 2013a. Data from: A phylogeny of birds based on over 1,500 loci collected by target enrichment and high-throughput sequencing. In: Dryad Data Repository.

McCormack JE, Harvey MG, Faircloth BC, Crawford NG, Glenn TC, Brumfield RT. 2013b. A phylogeny of birds based on over 1,500 loci collected by target enrichment and high-throughput sequencing. PLoS One 8:e54848.

Meiklejohn KA, Faircloth BC, Glenn TC, Kimball RT, Braun EL. 2016a. Analysis of a Rapid Evolutionary Radiation Using Ultraconserved Elements: Evidence for a Bias in Some Multispecies Coalescent Methods. Syst. Biol. 65:612-627.

Meiklejohn KA, Faircloth BC, Glenn TC, Kimball RT, Braun EL. 2016b. Data from: Analysis of a rapid evolutionary radiation using ultraconserved elements (UCEs): Evidence for a bias in some multi-species coalescent methods. In: Dryad.

Misof B, Liu S, Meusemann K, Peters RS, Donath A, Mayer C, Frandsen PB, Ware J, Flouri T, Beutel RG, et al. 2014a. Data from: Phylogenomics resolves the timing and pattern of insect evolution. In: Dryad Digital Repository.

Misof B, Liu S, Meusemann K, Peters RS, Donath A, Mayer C, Frandsen PB, Ware J, Flouri T, Beutel RG, et al. 2014b. Phylogenomics resolves the timing and pattern of insect evolution. Science 346:763-767.

Moyle RG, Oliveros CH, Andersen MJ, Hosner PA, Benz BW, Manthey JD, Travers SL, Brown RM, Faircloth BC. 2016a. Data from: Tectonic collision and uplift of Wallacea triggered the global songbird radiation. In: Dryad Data Repository.

Moyle RG, Oliveros CH, Andersen MJ, Hosner PA, Benz BW, Manthey JD, Travers SL, Brown RM, Faircloth BC. 2016b. Tectonic collision and uplift of Wallacea triggered the global songbird radiation. Nat Commun 7:12709.

Murray EA, Carmichael AE, Heraty JM. 2013a. Ancient host shifts followed by host conservatism in a group of ant parasitoids. Proc Biol Sci 280:20130495.

Murray EA, Carmichael AE, Heraty JM. 2013b. Data from: Ancient host shifts followed by host conservatism in a group of ant parasitoids. In: Dryad Data Repository.

Near TJ, Dornburg A, Eytan RI, Keck BP, Smith WL, Kuhn KL, Moore JA, Price SA, Burbrink FT, Friedman M, et al. 2013a. Data from: Phylogeny and tempo of diversification in the superradiation of spiny-rayed fishes. In: Dryad.

Near TJ, Dornburg A, Eytan RI, Keck BP, Smith WL, Kuhn KL, Moore JA, Price SA, Burbrink FT, Friedman M, et al. 2013b. Phylogeny and tempo of diversification

in the superradiation of spiny-rayed fishes. Proc Natl Acad Sci U S A 110:12738-12743.
Nguyen AD, Gotelli NJ, Cahan SH. 2016a. Data from: The evolution of heat shock protein sequences, cis-regulatory elements, and expression profiles in the eusocial Hymenoptera. In: Dryad.
Nguyen AD, Gotelli NJ, Cahan SH. 2016b. The evolution of heat shock protein sequences, cis-regulatory elements, and expression profiles in the eusocial Hymenoptera. BMC Evol. Biol. 16:15.
Oaks JR. 2011a. Data from: A time-calibrated species tree of Crocodylia reveals a recent radiation of the true crocodiles. In: Dryad Data Repository.
Oaks JR. 2011b. A time-calibrated species tree of Crocodylia reveals a recent radiation of the true crocodiles. Evolution 65:3285-3297.
Prebus M. 2017a. Data from: Insights into the evolution, biogeography and natural history of the acorn ants, genus Temnothorax Mayr (Hymenoptera: Formicidae). In: Dryad.
Prebus M. 2017b. Insights into the evolution, biogeography and natural history of the acorn ants, genus Temnothorax Mayr (hymenoptera: Formicidae). BMC Evol. Biol. 17:250.
Pyron RA, Wiens JJ. 2011. A large-scale phylogeny of Amphibia including over 2800 species, and a revised classification of extant frogs, salamanders, and caecilians. Mol Phylogenet Evol 61:543-583.
Pyron RA, Wiens JJ, Alexander Pyron R. 2011. Data from: A large-scale phylogeny of Amphibia including over 2800 species, and a revised classification of extant frogs, salamanders, and caecilians. In: Dryad.
Ran JH, Shen TT, Wang MM, Wang XQ. 2018a. Data from: Phylogenomics resolves the deep phylogeny of seed plants and indicates partial convergent or homoplastic evolution between Gnetales and angiosperms. In: Dryad Digital Repository.
Ran JH, Shen TT, Wang MM, Wang XQ. 2018b. Phylogenomics resolves the deep phylogeny of seed plants and indicates partial convergent or homoplastic evolution between Gnetales and angiosperms. Proc Biol Sci 285:20181012.
Reddy S, Kimball RT, Pandey A, Hosner PA, Braun MJ, Hackett SJ, Han K, Harshman J, Huddleston CJ, Kingston S, et al. 2017a. Data from: Why do phylogenomic data sets yield conflicting trees? Data type influences the avian tree of life more than taxon sampling. In: Dryad Digital Repository.
Reddy S, Kimball RT, Pandey A, Hosner PA, Braun MJ, Hackett SJ, Han KL, Harshman J, Huddleston CJ, Kingston S, et al. 2017b. Why Do Phylogenomic Data Sets

Yield Conflicting Trees? Data Type Influences the Avian Tree of Life more than Taxon Sampling. Syst. Biol. 66:857-879.

Richart CH, Hayashi CY, Hedin M. 2016a. Data from: Phylogenomic analyses resolve an ancient trichotomy at the base of Ischyropsalidoidea (Arachnida, Opiliones) despite high levels of gene tree conflict and unequal minority resolution frequencies. In: Dryad.

Richart CH, Hayashi CY, Hedin M. 2016b. Phylogenomic analyses resolve an ancient trichotomy at the base of Ischyropsalidoidea (Arachnida, Opiliones) despite high levels of gene tree conflict and unequal minority resolution frequencies. Mol Phylogenet Evol 95:171-182.

Rightmyer MG, Griswold T, Brady SG. 2013a. Data from: Phylogeny and systematics of the bee genus Osmia (Hymenoptera: Megachilidae) with emphasis on North American Melanosmia: subgenera, synonymies, and nesting biology revisited. In: Dryad Data Repository.

Rightmyer MG, Griswold T, Brady SG. 2013b. Phylogeny and systematics of the bee genus Osmia (Hymenoptera: Megachilidae) with emphasis on North American Melanosmia: subgenera, synonymies and nesting biology revisited. Syst. Entomol. 38:561-576.

Sauquet H, Ho SY, Gandolfo MA, Jordan GJ, Wilf P, Cantrill DJ, Bayly MJ, Bromham L, Brown GK, Carpenter RJ, et al. 2012. Testing the impact of calibration on molecular divergence times using a fossil-rich group: the case of Nothofagus (Fagales). Syst. Biol. 61:289-313.

Sauquet H, Ho SYW, Gandolfo MA, Jordan GJ, Wilf P, Cantrill DJ, Bayly MJ, Bromham L, Brown GK, Carpenter RJ, et al. 2011. Data from: Testing the impact of calibration on molecular divergence times using a fossil-rich group: the case of Nothofagus (Fagales). In: Dryad Data Repository.

Seago AE, Giorgi JA, Li J, Ślipiński A. 2011a. Data from: Phylogeny, classification and evolution of ladybird beetles (Coleoptera: Coccinellidae) based on simultaneous analysis of molecular and morphological data. In: Dryad Data Repository.

Seago AE, Giorgi JA, Li J, Ślipiński A. 2011b. Phylogeny, classification and evolution of ladybird beetles (Coleoptera: Coccinellidae) based on simultaneous analysis of molecular and morphological data. Mol. Phylogen. Evol. 60:137-151.

Sharanowski BJ, Dowling APG, Sharkey MJ. 2011a. Data from: Molecular phylogenetics of Braconidae (Hymenoptera: Ichneumonoidea) based on multiple nuclear genes and implications for classification. In: Dryad Data Repository.

Sharanowski BJ, Dowling APG, Sharkey MJ. 2011b. Molecular phylogenetics of Braconidae (Hymenoptera: Ichneumonoidea), based on multiple nuclear genes, and implications for classification. Syst. Entomol. 36:549-572.

Shen X-X. 2018. Data from: Tempo and mode of genome evolution in the budding yeast subphylum. In: Figshare.

Shen XX, Opulente DA, Kominek J, Zhou X, Steenwyk JL, Buh KV, Haase MAB, Wisecaver JH, Wang M, Doering DT, et al. 2018. Tempo and Mode of Genome Evolution in the Budding Yeast Subphylum. Cell 175:1533-1545 e1520.

Siler C, Brown RM, Oliveros CH, Santanen A. 2013. Data from: Multilocus phylogeny reveals unexpected diversification patterns in Asian Wolf Snakes (genus Lycodon). In: Dryad Data Repository.

Siler CD, Oliveros CH, Santanen A, Brown RM. 2013. Multilocus phylogeny reveals unexpected diversification patterns in Asian wolf snakes (genus Lycodon). Zool. Scr. 42:262-277.

Smith BT, Harvey MG, Faircloth BC, Glenn TC, Brumfield RT. 2014a. Data from: Target capture and massively parallel sequencing of ultraconserved elements for comparative studies at shallow evolutionary time scales. In: Dryad Digital Repository.

Smith BT, Harvey MG, Faircloth BC, Glenn TC, Brumfield RT. 2014b. Target capture and massively parallel sequencing of ultraconserved elements for comparative studies at shallow evolutionary time scales. Syst. Biol. 63:83-95.

Tolley KA, Townsend TM, Vences M. 2013a. Data from: Large-scale phylogeny of chameleons suggests African origins and Eocene diversification. In: Dryad Data Repository.

Tolley KA, Townsend TM, Vences M. 2013b. Large-scale phylogeny of chameleons suggests African origins and Eocene diversification. Proc Biol Sci 280:20130184.

Unmack PJ, Allen GR, Johnson JB. 2013a. Data from: Phylogeny and biogeography of rainbowfishes (Melanotaeniidae) from Australia and New Guinea. In: Dryad Data Repository.

Unmack PJ, Allen GR, Johnson JB. 2013b. Phylogeny and biogeography of rainbowfishes (Melanotaeniidae) from Australia and New Guinea. Mol Phylogenet Evol 67:15-27.

Varga T, Krizsán K, Földi C, Dima B, Sánchez-García M, Sánchez-Ramírez S, Szöllősi GJ, Szarkándi JG, Papp V, Albert L, et al. 2019a. Data from: Megaphylogeny resolves global patterns of mushroom evolution. In: Dryad.

Varga T, Krizsán K, Földi C, Dima B, Sánchez-García M, Sánchez-Ramírez S, Szöllősi GJ, Szarkándi JG, Papp V, Albert L, et al. 2019b. Megaphylogeny resolves global patterns of mushroom evolution. Nat Ecol Evol 3:668-678.

Wainwright PC, Smith WL, Price SA, Tang KL, Sparks JS, Ferry LA, Kuhn KL, Near TJ. 2012b. Data from: The evolution of pharyngognathy: a phylogenetic and functional appraisal of the pharyngeal jaw key innovation in labroid fishes and beyond. In: Dryad Data Repository.

Wainwright PC, Smith WL, Price SA, Tang KL, Sparks JS, Ferry LA, Kuhn KL, Near TJ, Eytan RI. 2012a. The evolution of pharyngognathy: a phylogenetic and functional appraisal of the pharyngeal jaw key innovation in labroid fishes and beyond. Syst. Biol. 61:1001-1027.

Whelan NV, Kocot KM, Moroz TP, Mukherjee K, Williams P, Paulay G, Moroz LL, Halanych KM. 2017a. Author Correction: Ctenophore relationships and their placement as the sister group to all other animals. Nat Ecol Evol 1:1783.

Whelan NV, Kocot KM, Moroz TP, Mukherjee K, Williams P, Paulay G, Moroz LL, Halanych KM. 2017b. Data from: Ctenophora Phylogeny Datasets and Core Orthologs. In: Figshare.

Wood HM, Matzke NJ, Gillespie RG, Griswold CE. 2012. Data from: Treating fossils as terminal taxa in divergence time estimation reveals ancient vicariance patterns in the palpimanoid spiders. In: Dryad Data Repository.

Wood HM, Matzke NJ, Gillespie RG, Griswold CE. 2013. Treating fossils as terminal taxa in divergence time estimation reveals ancient vicariance patterns in the palpimanoid spiders. Syst. Biol. 62:264-284.

Worobey M, Han G, Rambaut A. 2014a. Data from: A synchronized global sweep of the internal genes of modern avian influenza virus. In: Dryad Data Repository.

Worobey M, Han GZ, Rambaut A. 2014b. A synchronized global sweep of the internal genes of modern avian influenza virus. Nature 508:254-257.

Wu S, Edwards S, Liu L. 2019. Data from: Genome-scale DNA sequence data and the evolutionary history of placental mammals. In: Figshare.

Wu S, Edwards S, Liu L. 2018. Genome-scale DNA sequence data and the evolutionary history of placental mammals. Data Brief 18:1972-1975.
